# Evolution of biofilm-adapted gene expression profiles in *lasR*-deficient clinical *Pseudomonas aeruginosa* isolates

**DOI:** 10.1038/s41522-022-00268-1

**Published:** 2022-02-14

**Authors:** Alexander Jeske, Alejandro Arce-Rodriguez, Janne G. Thöming, Jürgen Tomasch, Susanne Häussler

**Affiliations:** 1 Department of Molecular Bacteriology, Helmholtz Centre for Infection Research, 38124 Braunschweig, Germany; 2 Institute for Molecular Bacteriology, TWINCORE, Centre for Experimental and Clinical Infection Research, 30265 Hannover, Germany; 3 Department of Clinical Microbiology, Copenhagen University Hospital—Rigshospitalet, 2100 Copenhagen, Denmark; 4 Cluster of Excellence RESIST (EXC 2155), Hannover Medical School, 30265 Hannover, Germany

**Keywords:** Pathogens, Health care

## Abstract

The overall success of a pathogenic microbe depends on its ability to efficiently adapt to challenging conditions in the human host. Long-term evolution experiments track and predict adaptive trajectories and have contributed significantly to our understanding of the driving forces of bacterial adaptation. In this study, we conducted a cross-sectional study instead of long-term longitudinal evolution experiments. We analyzed the transcriptional profiles as well as genomic sequence variations of a large number of clinical *Pseudomonas aeruginosa* isolates that have been recovered from different infected human sites. Convergent changes in gene expression patterns were found in different groups of clinical isolates. The majority of repeatedly observed expression patterns could be attributed to a defective *lasR* gene, which encodes the major quorum-sensing regulator LasR. Strikingly, the gene expression pattern of the *lasR*-defective strains appeared to reflect a transcriptional response that evolves in a direction consistent with growth within a biofilm. In a process of genetic assimilation, *lasR*-deficient *P. aeruginosa* isolates appear to constitutively express a biofilm-adapted transcriptional profile and no longer require a respective environmental trigger. Our results demonstrate that profiling the functional consequences of pathoadaptive mutations in clinical isolates reveals long-term evolutionary pathways and may explain the success of *lasR* mutants in the opportunistic pathogen *P. aeruginosa* in a clinical context.

## Introduction

Thanks to advances in sequencing technology, it is possible to analyze the transcriptomes of many bacterial isolates with little effort, at a low cost, and within a short period of time. RNA sequencing data offer the potential for large-scale comparative studies, which aim at deciphering coordinated regulation of genes within an organism. Thus, new insights into complex bacterial adaptation strategies, for example during an infection process, can be achieved^1^.

The Gram-negative, opportunistic pathogen *Pseudomonas aeruginosa* is considered one of the most important pathogens involved in nosocomial infections^2^. The bacterium causes high morbidity and mortality, especially in patients with weakened immune systems and burn victims^3^. In cystic fibrosis (CF) patients, lifelong chronic *P. aeruginosa* infections lead to an over-activation of the immune system and to extensive tissue damage in the patients’ lungs^4–6^. In the course of these long-term infections, *P. aeruginosa* not only adapts by orchestrating the transcription of complex regulatory gene networks, but the bacteria also develop a number of pathoadaptive mutations that promote survival in the harsh environment of the human host^7–10^. The adapted phenotypes have fitness advantages over the originally infecting wild-type strain, e.g., by escaping immune recognition, saving energy by using public goods or developing resistance to antimicrobial agents^4,7,11,12^. Understanding the evolutionary trajectories that *P. aeruginosa* follows during long-term infections is an important task in order to optimize treatment and predict the clinical course of the infection^13–15^.

In this study, we analyzed the genomes and transcriptomes of a large number of clinical *P. aeruginosa* isolates previously recorded under both planktonic and biofilm growth conditions^14,16,17^. We found that complex gene expression patterns evolved independently in different clinical *P. aeruginosa* isolates and that a majority of the observed expression patterns could be attributed to a defective *lasR* gene, encoding the major quorum sensing (QS) regulator LasR^18,19^. Strikingly, whereas a great number of genes were differentially regulated in the *lasR*-proficient isolates upon shifting from planktonic to biofilm growth conditions, the *lasR*-defective isolates exhibited a transcriptional signature under planktonic culture conditions that already resembled a biofilm transcriptional profile. Our results demonstrate that the analysis of transcriptomic and genomic data from a large variety of clinical isolates provides new insights into how adaptive mutations drive gene expression programs and regulate phenotypic outcomes responsible for bacterial adaptation to altered and challenging host niche habitats.

## Results

### Distribution of single gene expression values across clinical *P. aeruginosa* isolates

**Fig. 1:**
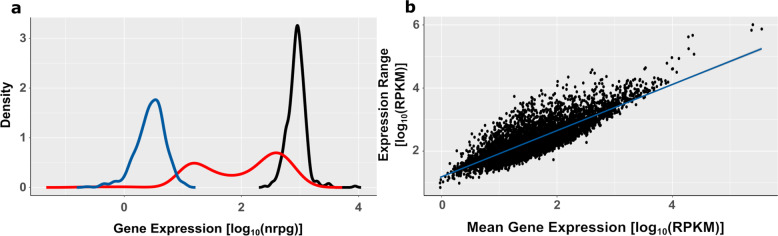
Distribution of gene expression profiles across 414 clinical *P. aeruginosa* isolates. **a** The distribution of the expression values is shown as an example for the three genes *cobK* (blue, low expression), *sahH* (black, high expression), and *mexY* (red, bi-modal expression) across the 414 clinical isolates. Distribution was calculated using the density() function in R with nrpg values as input. **b** Mean gene expression values relative to the expression value range of all tested isolates. Expression values were normalized for gene length (RPKM). Blue line = linear regression; adjusted *R*^2^ = 0.7.

We have previously transcriptionally profiled a collection of 414 clinical *P. aeruginosa* isolates following growth in lysogeny broth (LB) media until the early stationary phase (OD_600_ of 2)^16,17,20^. Here, we re-analyzed the data and recorded the distribution of individual gene expression values across all 414 clinical isolates. As exemplified in Fig. 1a, we identified genes that were expressed at high levels in most isolates, while other genes had overall low expression levels. In general, it appears that the higher the overall expression level of a particular gene, the higher the variation in expression level between the different clinical isolates (Fig. 1b). We also observed that some genes showed a much greater variation in their expression values, with some isolates having lower levels and others having higher levels. This at least bi-modal gene expression pattern is exemplified in Fig. 1a.

**Fig. 2:**
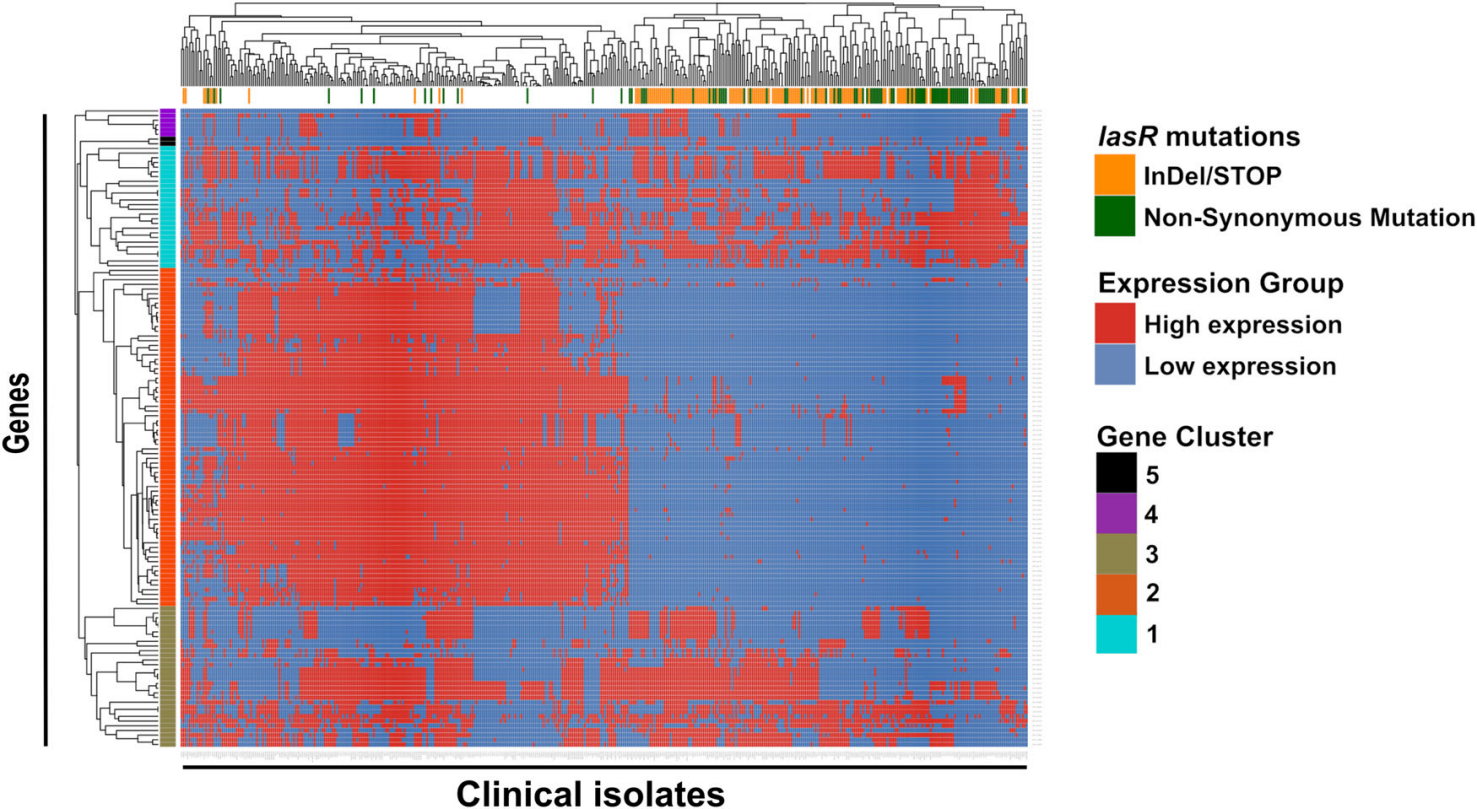
Distribution of bi-modal gene expression across 414 clinical *P. aeruginosa* isolates. Multimodal (R-package ‘multimode’, adjusted *p*-value ≤ 0.1) gene expression distributions were recorded, and the distribution of the 136 bi-modal genes (*y*-axis), which showed high expression (red) or a low expression (blue) across the 414 clinical isolates (*x*-axis), was recorded. The resulting binary matrix was used as a basis for a heat map (binary clustering of rows and columns). Clinical isolates harboring non-synonymous mutations in *lasR* are marked in green, insertions/deletions/premature stop codons (InDel/STOP) are marked in yellow. Clustering uncovered 5 groups of genes with a similar bi-modal expression pattern in the clinical isolates. They were colored on the *y*-axis for a better overview (Gene Cluster 1-5). Only bi-modally expressed genes that are assigned in both reference strains, PA14 and PAO1 were included.

We identified overall 136 genes with a bi-modal gene expression distribution by the use of an algorithm that distinguishes between uni- and at least bi-modality expressed genes (Supplementary Data 1, in some cases even a tri-modal distribution could be seen). We then classified the isolates into either a high- or a low-expression group with respect to each of the 136 at least bi-modal genes (distributions and cut-off visualization in Supplementary Fig. 7). The resulting binary dataset was clustered in a heat-map and we identified five gene clusters containing genes with either high or low expression patterns in the individual clinical isolates (Fig. 2). The largest cluster contained 76 genes (Fig. 2; gene cluster 2). High or low expression of those genes was identified as the main driver of the clustering of the individual clinical isolates into two major groups (Fig. 2). In this gene cluster 2, we found a significant enrichment of genes belonging to the KEGG pathway functional categories “phenazine biosynthesis”, “biofilm formation”, and “quorum sensing” (Supplementary Data 1).

As many of the genes of these functional categories are under the control of the major QS regulator LasR, we evaluated whether mutations of the *lasR* gene could explain the distinct expression patterns of these at least bi-modally distributed genes. For this purpose, we analyzed previously published whole-genome sequencing data of the isolates^16^ and evaluated the *lasR* allele status in the 414 clinical isolates. In addition to gene-inactivating mutations due to frameshifts (insertion or deletions, summarized as InDels) or pre-mature stop codons (STOP), there were also non-silent mutations that led to changes in the amino acid sequence of LasR. As depicted in Supplementary Fig. 1, amino acid exchanges were found throughout the entire protein sequence. Nevertheless, there were marked hotspots in the N-terminal and C-terminal region, which are involved in dimerization and DNA binding, respectively^21,22^, indicating that they might result in the functional inactivation of LasR^23^.

There was a clear correlation between the expression status of bi-modal genes and the allele status of the transcriptional regulator LasR (Fig. 2; yellow and green squares). This indicates that variations in the expression pattern of a majority of the bi-modally expressed genes across the clinical isolates were associated with the presence/absence of a functional *lasR* gene and underscores the dominant role of LasR in shaping the transcriptional profile in our collection of clinical isolates. We did not identify large groups of clinical isolates that exhibited a characteristic expression pattern of genes belonging to the two other major clusters (Fig. 2; gene clusters 1 and 3). This suggests that there does not seem to be another major regulatory gene (in addition to *lasR*) that influences a comparably large fraction of the bi-modally expressed genes. Nevertheless, future work should concentrate on the identification of additional genes, which influence the expression of smaller groups of bi-modal genes.

### Identification of the core *lasR* regulon

Since LasR shapes the transcriptional profile of a large fraction of our clinical isolates, we aimed for the identification of genes that were differentially regulated in the group of clinical isolates that expressed a wild-type *lasR* allele versus the group of isolates harboring a non-functional *lasR*. We concentrated on 28 randomly selected clinical isolates with *lasR* alleles exhibiting neither synonymous nor non-synonymous sequence variations in the *lasR* gene (as compared to the PA14 or PAO1 *lasR* gene) and 21 clinical isolates with *lasR* inactivating (InDels/stop) mutant alleles (hereinafter described as *lasR*^*WT*^ and lasR* isolates, respectively). Both, planktonic and biofilm transcriptomes, were available for those 49 clinical isolates. We then calculated the differential gene expression between the *lasR*^*WT*^ and lasR* group. In total, 722 genes were differentially regulated (corrected *p*-value ≤ 0.05 and log_2_-fold change |log_2_FC | ≥ 1). Of those, 412 genes were up- and 310 genes were downregulated in lasR* isolates compared to *lasR*^*WT*^ isolates (Supplementary Data 2).

As expected, inactivation of *lasR* in the lasR* isolates led to a reduced expression of genes encoding for important virulence factors, such as phenazines (*phzA-G*), the protease LasA (*lasA*), the elastase LasB (*lasB*) or rhamnolipids (*rhlAB*). Accordingly, we identified genes belonging to the KEGG^24^ pathways of “phenazine biosynthesis”, “quorum sensing”, and “biofilm formation” as being significantly enriched (adjusted *p-*value ≤ 0.05, hypergeometric test) in the group of genes that were differentially expressed between the clinical *lasR*^*WT*^ and lasR* isolates. We next phenotypically characterized a random selection of *lasR*^*WT*^ (*n* = 10) and lasR* isolates (*n* = 8). In agreement with the finding that LasR governs the production of a range of virulence factors, we found a significantly reduced virulence of the lasR* isolates in a *Galleria mellonella* infection model compared to the isolates with functional LasR (Supplementary Fig. 2a). In addition, the lasR* isolates showed a significantly reduced protease and elastase production following growth in planktonic cultures (Supplementary Fig. 2b, c).

**Fig. 3:**
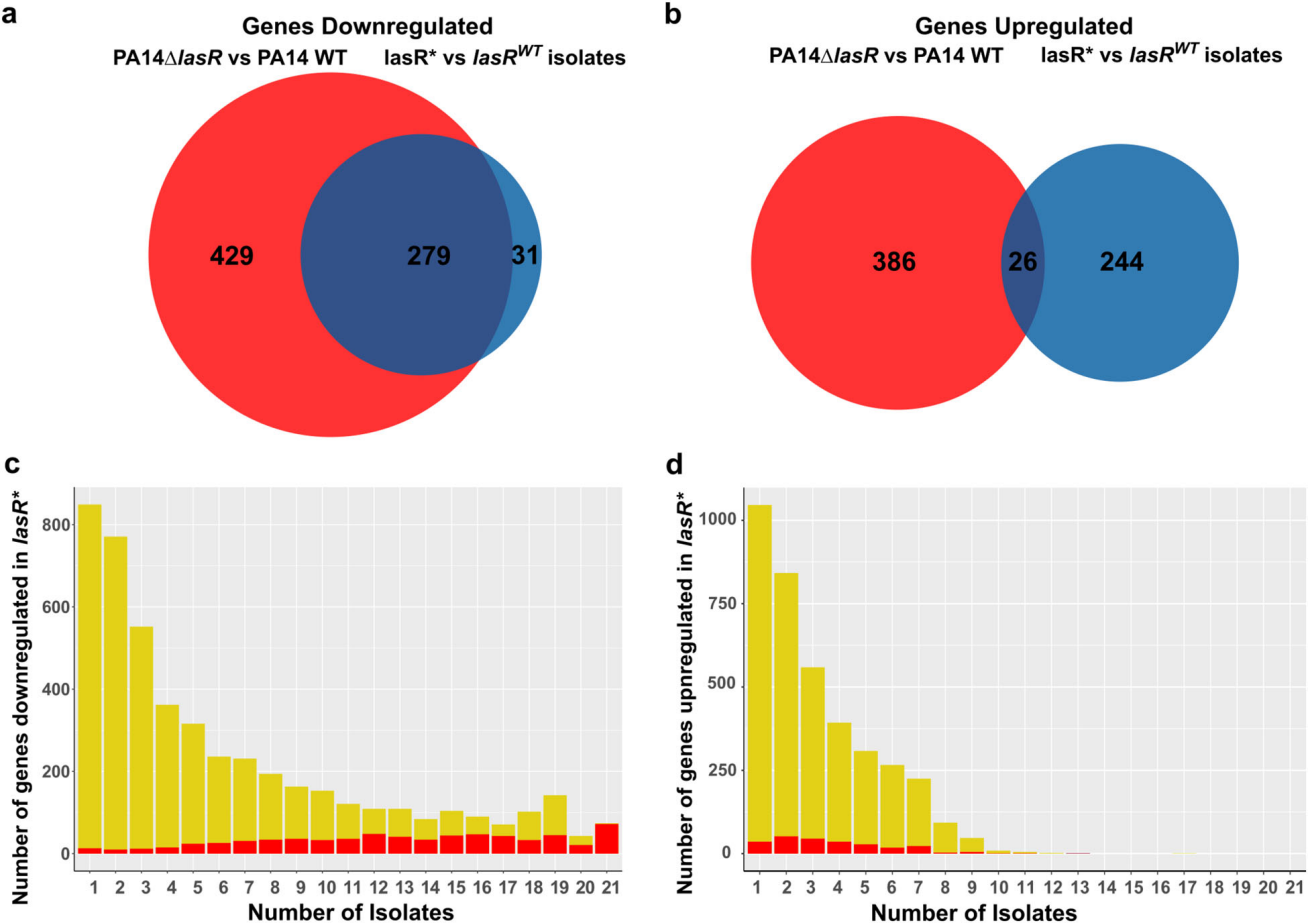
The LasR regulon. Venn diagrams showing the overlap between genes that are downregulated (**a**) or upregulated (**b**) in lasR* as compared to *lasR*^*WT*^ isolates (21 lasR* vs. 28 *lasR*^*WT*^ isolates; blue) and the genes that are regulated respectively in PA14 ∆*lasR* as compared to PA14 WT (3 biological replicates; red) (log_2_FC > 1, *p* value < 0.05). Number of genes that were commonly (log_2_FC > 1) downregulated (**c**) or upregulated (**d**) in 1, 2, 3,…21 clinical lasR* isolates compared to all analyzed *lasR*^*WT*^ isolates recorded (yellow bars). The proportion of genes that was identified to be also regulated in the constructed PA14 ∆*lasR* (compared to PA14 WT) is marked in red.

In order to further elucidate on the LasR regulon, we recorded the transcriptional profile of the reference strain UCBPP-PA14 (PA14 WT^25^) as compared to a clean *lasR* deletion mutant (PA14 ∆*lasR*^26^) under the same growth conditions as applied for the clinical isolates (Supplementary Data 3). Of the genes that were significantly downregulated in the lasR* versus *lasR*^*WT*^ isolates, 90% were also significantly downregulated in PA14 ∆*lasR* vs. PA14 WT (Fig. 3a).

In order to identify strain-specific as well as common transcriptional signatures associated with *lasR* deficiency, we calculated log_2_FCs of each of the 21 lasR* isolates against the group of *lasR*^*WT*^ isolates. Totally, 1202 individual genes were down-regulated (log_2_FC ≤ −1) in at least 10 lasR* isolates and 74 genes were downregulated in all 21 lasR* isolates as compared to the group of *lasR*^*WT*^ isolates (Supplementary Data 6). Of note, 72 of those 74 genes that were commonly downregulated across all 21 clinical lasR* isolates, were also identified as being part of the *lasR* regulon in the PA14 WT strain background (Fig. 3).

Our results clearly indicate that there is a core gene set, which is expressed in a *lasR*-dependent manner across many different *P. aeruginosa* isolates thus underscoring the value of a transcriptional profiling approach of multiple clinical isolates.

The complementary analysis of *lasR*-dependent gene expression in clinical isolates as well as in a targeted engineered reference strain allowed us to define the *lasR* core regulon, which consists of overall 138 genes (Table 1). Only those genes were included, which were downregulated in at least 90% (19 out of 21) of the analyzed single clinical lasR* isolates compared to the *lasR*^*WT*^ isolates (*n* = 28), and which were also significantly downregulated in PA14 ∆*lasR* as compared to its PA14 wild-type.

**Table 1:** The *lasR* core regulon.

Locus Tag	Gene Name	Locus Tag	Gene Name	Locus Tag	Gene Name
PA14_00650	*–*	PA14_21000	*–*	PA14_40310	*–*
PA14_01490	–	PA14_21010	*–*	PA14_41500	*–*
PA14_01760	*nuh*	PA14_21020	*–*	PA14_42910	*–*
PA14_02220	–	PA14_21030	*–*	PA14_42940	*–*
PA14_07430	*–*	PA14_26020	*–*	PA14_42950	*–*
PA14_09400	*phzS*	PA14_30570	*potF*	PA14_42960	*–*
PA14_09410	*phzG1*	PA14_30580	*–*	PA14_42970	*–*
PA14_09420	*phzF1*	PA14_30620	*–*	PA14_42980	*–*
PA14_09440	*phzE1*	PA14_30630	*pqsH*	PA14_42990	*–*
PA14_09450	*phzD1*	PA14_31150	*–*	PA14_43000	*–*
PA14_09460	*phzC1*	PA14_31290	*pa1L*	PA14_43020	*–*
PA14_09470	*phzB1*	PA14_31360	*–*	PA14_43030	*–*
PA14_09480	*phzA1*	PA14_32590	*dipZ2*	PA14_43040	*–*
PA14_09490	*phzM*	PA14_32600	*–*	PA14_43050	*–*
PA14_09700	*pqsL*	PA14_32610	*dsbG*	PA14_43090	*–*
PA14_09900	*prpL*	PA14_33290	*–*	PA14_45940	*lasI*
PA14_10360	*–*	PA14_33450	*treA*	PA14_45950	*rsaL*
PA14_10380	*–*	PA14_34020	*–*	PA14_46510	*–*
PA14_10490	*–*	PA14_34330	*–*	PA14_46520	*–*
PA14_10500	*ccoN*	PA14_34810	*mxaA*	PA14_46530	*–*
PA14_10530	*–*	PA14_34820	*–*	PA14_46540	*–*
PA14_10540	*fixG*	PA14_34830	*–*	PA14_46550	*–*
PA14_10550	*cysI*	PA14_34840	*–*	PA14_48040	*aprI*
PA14_10560	*–*	PA14_34870	*chiC*	PA14_48060	*aprA*
PA14_13360	*–*	PA14_36310	*hcnC*	PA14_48090	*aprF*
PA14_13370	*–*	PA14_36320	*hcnB*	PA14_48100	*aprE*
PA14_13380	*–*	PA14_36330	*hcnA*	PA14_48115	*aprD*
PA14_13390	*–*	PA14_36530	*–*	PA14_48140	*aprX*
PA14_15090	*-*	PA14_36620	*–*	PA14_49260	*napB*
PA14_16250	*lasB*	PA14_36820	*–*	PA14_49310	*–*
PA14_18630	*eprS*	PA14_37745	*–*	PA14_51350	*phnB*
PA14_19100	*rhlA*	PA14_37760	*–*	PA14_51380	*pqsE*
PA14_19110	*rhlB*	PA14_37770	*–*	PA14_51390	*pqsD*
PA14_19120	*rhlR*	PA14_37780	*–*	PA14_51410	*pqsC*
PA14_19130	*rhlI*	PA14_38260	*–*	PA14_51420	*pqsB*
PA14_19870	*ldh*	PA14_39880	*phzG2*	PA14_51430	*pqsA*
PA14_19900	*–*	PA14_39890	*phzF2*	PA14_53250	*cpbD*
PA14_19910	*pdhB*	PA14_39910	*phzE2*	PA14_55080	*–*
PA14_20610	*lecB*	PA14_39925	*phzD2*	PA14_55940	*–*
PA14_20900	*–*	PA14_39945	*phzC2*	PA14_60750	*pra*
PA14_20920	*–*	PA14_39960	*phzB2*	PA14_61870	*–*
PA14_20940	*-*	PA14_39970	*phzA2*	PA14_63170	*cueR*
PA14_20950	*fabH2*	PA14_40180	*–*	PA14_66840	*phaC2*
PA14_20960	*–*	PA14_40200	*–*	PA14_68930	*–*
PA14_20970	*cyp23*	PA14_40290	*lasA*	PA14_68940	*–*
PA14_20980	*–*	PA14_40300	*–*	PA14_69560	*hcpB*

The core regulon was defined as the (*n* = 138) genes that showed a log_2_FC ≤ −1 (corrected *p*-value ≤ 0.05) in at least 19 of overall 21 lasR* compared to 28 *lasR*^*WT*^ isolates and were significantly downregulated (log_2_FC ≤ −1) in PA14 ∆*lasR* compared to PA14 WT. Cells were grown in LB at 37 °C and 180 rpm shaking until an OD_600_ = 2.

### Upregulation of gene expression in clinical isolates exhibiting a non-functional LasR

It is well established that LasR is a transcriptional activator that governs a large QS regulon^27^. However, we also found that *lasR* mutations in the clinical isolates led to an increased expression of certain genes when compared to *lasR*^*WT*^ isolates (Fig. 3b). Table 2 lists a selection of 123 genes (organized in operon structures) of distinct functional categories that were expressed at higher levels in the absence of a functional *lasR* (full gene list in Supplementary Data 2). The majority of those genes (*n* = 57) are associated with the degradation of complex carbon sources, such as aromatic compounds or lipids. Another 21 genes are involved in various mechanisms of iron acquisition (such as *hasRDE* and *pvdGEFN*), and the *kdpABC* operon is associated with sensing low extracellular potassium levels. In addition, 12 genes were upregulated in the lasR* isolates that encoded for *cup* fimbriae or the biosynthesis of the *pel* exopolysaccharide. Moreover, the spermidine transport system encoding *potABCD* exhibited increased expression levels in lasR* isolates compared to *lasR*^*WT*^ isolates. Furthermore, acquisition of extracellular phosphate either via the *phn* transport system, the (extracellular) degradation of phosphatidylcholine (*glpQ* and *glpD*), or the type 2 secretion system (T2SS, *hxc*) seems to be pronounced in the lasR* isolates. Interestingly, also multiple resistance genes including the multi-drug efflux pump *mexCD-oprJ* were significantly upregulated in lasR* isolates.

**Table 2:** Selection of upregulated genes in lasR* isolates under planktonic growth conditions as compared to *lasR*^*WT*^ isolates. For a complete list of regulated genes see Supplementary Data 2.

Locus Tag	Gene Name	log_2_ FC	Locus Tag	Gene Name	log_2_ FC	Locus Tag	Gene Name	log_2_ FC
*Metabolism and transport of carbon sources*						*Iron acquisition*		
PA14_02760	*catI*	1.41	PA14_34260	*metI*	1.42	PA14_01870	*–*	1.07
PA14_04550	*glpQ*	1.13	PA14_34270	*metN*	1.42	PA14_02410	*–*	1.29
PA14_09980	*dkgB*	1.51	PA14_34280	*metQ*	1.55	PA14_06160	*fiuA*	1.04
PA14_10590	*hpcG*	1.01	PA14_34360	*mtlD*	1.18	PA14_09970	*fpvB*	1.05
PA14_10600	*hpaX*	1.48	PA14_34370	*mtlK*	0.86	PA14_10170	*fepB*	1.39
PA14_10610	*hpcD*	1.08	PA14_34390	*mtlG*	1.45	PA14_13430	*fecA*	1.15
PA14_10630	*hpcC*	0.87	PA14_34410	*mtlF*	1.48	PA14_20010	*hasR*	1.46
PA14_10640	*hpaG2*	1.07	PA14_34420	*mtlE*	1.01	PA14_20030	*hasD*	1.13
PA14_10650	*hpaG1*	1.23	PA14_42080	*fadB*	1.00	PA14_20040	*hasE*	1.29
PA14_10900	*ydjL*	1.55	PA14_43420	*–*	1.18	PA14_32740	*optS*	1.20
PA14_10990	*hpaC*	0.92	PA14_45000	*gcl*	1.45	PA14_33270	*pvdG*	1.32
PA14_11000	*hpaA*	0.96	PA14_45010	*hyi*	1.37	PA14_33690	*pvdE*	2.51
PA14_11020	*fabG*	1.40	PA14_45020	*glxR*	1.07	PA14_33700	*pvdF*	1.45
PA14_17610	*potD*	1.22	PA14_45030	*ttuD*	1.46	PA14_33720	*pvdN*	1.66
PA14_17620	*potC*	1.41	PA14_45050	*pykF*	1.41	PA14_37730	*–*	1.63
PA14_17630	*potB*	1.26	PA14_47920	*–*	1.28	PA14_33740	*pvdP*	0.96
PA14_17640	*potA*	1.34	PA14_47930	*–*	1.10	PA14_37900	*–*	1.49
PA14_17930	*glpD*	1.27	PA14_47940	*–*	1.46	PA14_39650	*cirA*	3.77
PA14_23160	*gltS*	1.35	PA14_47960	*–*	1.09	PA14_39820	*ufrA*	1.43
PA14_23170	*hutG*	1.17	PA14_48000	*–*	1.29	PA14_55340	*exbD2*	1.52
PA14_23190	*–*	1.09	PA14_52810	*dctM*	1.06	PA14_55360	*exbB2*	1.06
PA14_26210	*hisP*	1.14	PA14_52820	*dctQ*	1.39			
PA14_26220	*hisM*	1.33	PA14_52840	*dctP*	1.06	*Phosphate assimilation/T2SS*		
PA14_26230	*hisQ*	1.71	PA14_52900	*–*	1.21	PA14_20300	*phnC*	1.39
PA14_26240	*hisJ*	1.18	PA14_63620	*lipC*	1.21	PA14_20320	*phnD*	1.57
PA14_26260	*–*	1.14	PA14_63640	*fadH2*	1.64	PA14_20330	*phnE*	1.58
PA14_32080	*xylX*	1.50	PA14_64800	*vanA*	2.57	PA14_20370	*phnH*	1.75
PA14_32100	*xylY*	1.50	PA14_64810	*vanB*	1.72	PA14_20380	*phnI*	1.46
			PA14_65850	*rocE*	1.32	PA14_20390	*phnJ*	1.65
						PA14_20400	*phnK*	1.35
*Attachment*			*Resistance*			PA14_21110	*plcN*	1.00
PA14_11070	*cupB2*	1.93	PA14_18760	*mexP*	1.28	PA14_21410	*phoA*	1.37
PA14_11080	*cupB3*	1.29	PA14_18780	*mexQ*	1.22	PA14_21620	*oprP*	1.53
PA14_11090	*cupB4*	1.35	PA14_18790	*opmE*	1.20	PA14_31620	*-*	1.40
PA14_11100	*cupB5*	0.97	PA14_32270	*oprD3*	1.71	PA14_47300	*phnW*	0.85
PA14_11110	*cupB6*	1.01	PA14_60820	*oprJ*	1.26	PA14_55440	*hxcR*	1.27
PA14_35390	*pvcC*	1.00	PA14_60830	*mexD*	1.45	PA14_55450	*hxcQ*	1.39
PA14_35420	*pvcB*	1.04	PA14_60850	*mexC*	1.54	PA14_55460	*hxcZ*	1.79
PA14_35430	*pvcA*	1.61	PA14_73040	*amiA*	2.47	PA14_55490	*hxcT*	0.83
PA14_36990	*-*	1.14				PA14_55500	*hxcV*	1.11
PA14_37000	*cupA5*	1.45						
PA14_37010	*cupA4*	1.31	*Spermidine transport*			*Potassium sensing*		
PA14_37030	*cupA3*	1.37	PA14_17610	*potD*	1.22	PA14_43370	*kdpC*	1.07
PA14_37040	*cupA2*	1.26	PA14_17620	*potC*	1.41	PA14_43380	*kdpB*	1.72
PA14_51460	*cupC2*	1.57	PA14_17630	*potB*	1.26	PA14_43400	*kdpA*	1.84
			PA14_17640	*potA*	1.34	PA14_43405	*kdbF*	2.34
*Exopolysaccharide synthesis*								
PA14_24480	*pelA*	0.85						
PA14_24550	*pelF*	1.18						
PA14_24560	*pelG*	1.26						

In contrast to the common set of genes that were consistently downregulated in the majority of the lasR* isolates (Fig. 3c), there were by far fewer genes that were commonly upregulated across the different lasR* isolates. As depicted in Fig. 3d, only one gene (PA14_33800) of the overall 3797 genes that reached the log_2_FC threshold in at least one clinical isolate was upregulated in a maximum of 17 clinical lasR* isolates, and only 18 genes were upregulated in at least 10 of the 21 clinical lasR* isolates. This indicates that an upregulation of genes in a *lasR*-deficient background is to a much higher degree dependent on the individual characteristics of a strain. In line with this, the overlap between the genes that were upregulated in both, PA14 ∆*lasR* and the lasR* clinical isolates, was much smaller as compared to the genes that were commonly downregulated (Fig. 3b).

### LasR becomes less important under biofilm growth conditions

**Fig. 4:**
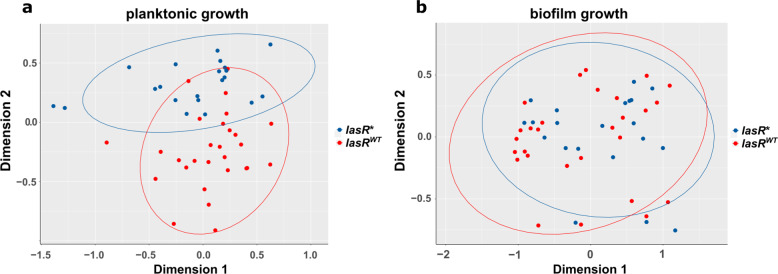
Multidimensional scaling plots based on the transcriptomes of 21 clinical lasR* and 28 *lasR*^*WT*^*P. aeruginosa* isolates under planktonic and biofilm growth conditions. For transcriptional profiling, bacteria were grown in rich LB media until an OD_600_ of 2 at 37 °C and 180 rpm for planktonic growth (**a**), or statically in biofilms for 48 h (**b**). Reads were normalized for the individual library size and genes with coverage below 1 count per million were excluded from the analysis. Ellipses display the 95% confidence intervals.

In addition to the transcriptional profiles of the lasR* (*n* = 21) and *lasR*^*WT*^ (*n* = 28) isolates under planktonic growth conditions, we also recorded their transcriptomes under biofilm growth conditions^14^. While the planktonic gene expression profiles of the two groups of clinical isolates were clearly separated in a multi-dimensional scaling plot (MDS), their transcriptomes did not cluster separately when the isolates were grown under biofilm growth conditions (Fig. 4).

In accordance, only 53 genes (25 upregulated and 28 downregulated, Supplementary Data 4) showed a differential expression in the lasR* isolates (compared to *lasR*^*WT*^) under biofilm growth conditions (as compared to 722 genes under planktonic conditions). We also recorded the transcriptional profiles of PA14 WT and PA14 ∆*lasR* under biofilm growth conditions (Supplementary Data 5). Again, we detected a reduction (by approx. 20%) in the overall number of differentially regulated genes between PA14 WT and PA14 ∆*lasR* under biofilm growth conditions (780 and 978 genes were differentially regulated under biofilm and planktonic growth conditions, respectively, Supplementary Fig. 3).

Our finding that the differential gene expression in clinical isolates harboring a non-functional *lasR* gene, can be alleviated under biofilm growth conditions indicates that the activity of LasR becomes less important under biofilm growth conditions. We also found that genes of the pyocyanin biosynthesis gene cluster (*phzG, phzF*) were expressed at even higher levels in the lasR* isolates under biofilm growth conditions (Supplementary Data 4). This demonstrates that despite the finding that they belong to the core *lasR* regulon under planktonic growth conditions, they are not governed by LasR under biofilm growth conditions. Instead, there seem to be alternative ways to induce the phenazine biosynthesis genes under biofilm growth conditions that are even more efficient. Our finding is in accordance with previous reports on a strong pyocyanin production of late stationary phase-grown *lasR* mutants^28^.

### Virulence factor expression becomes independent of *lasR* but not of *rhlR* under biofilm growth conditions

**Fig. 5:**
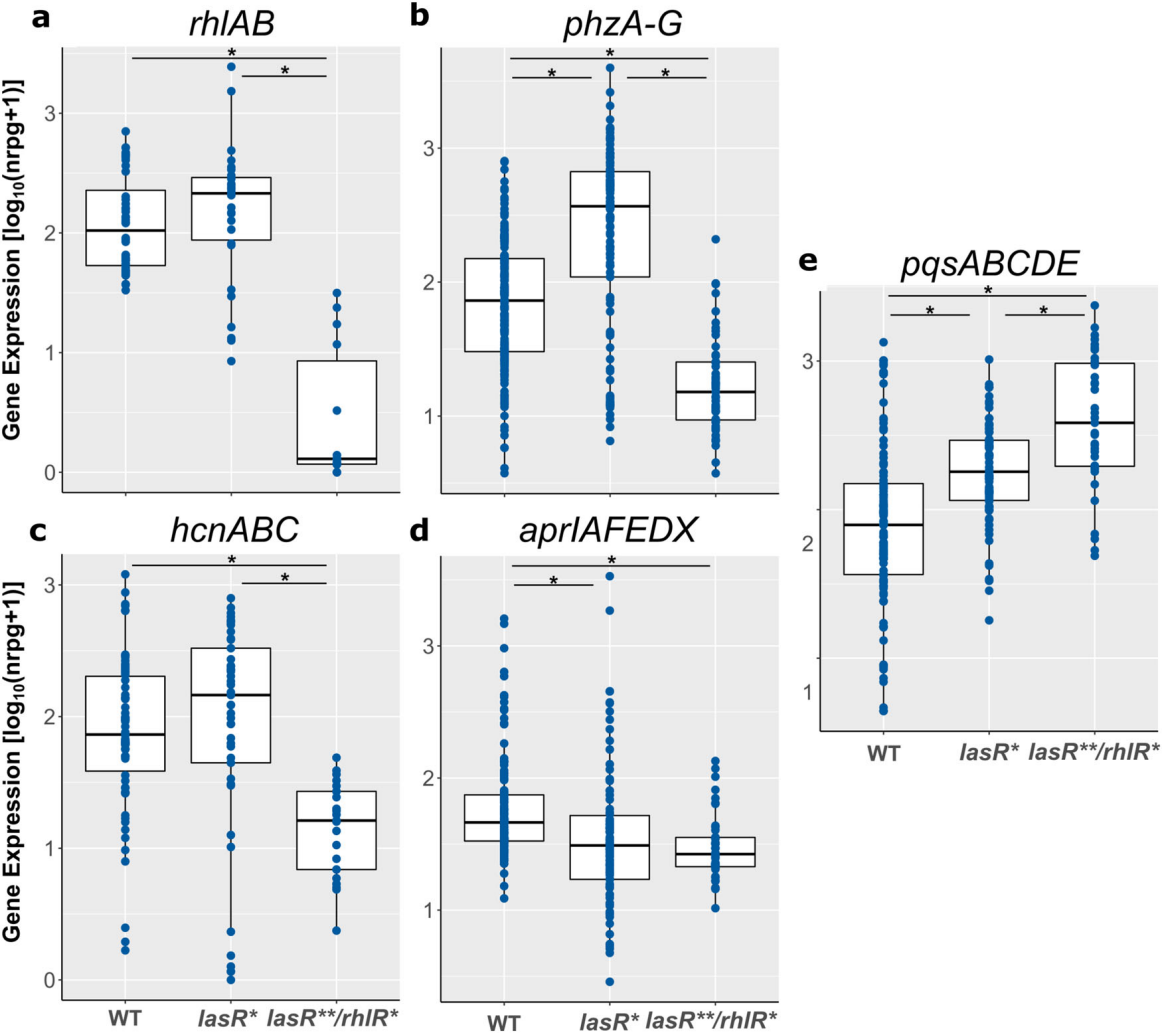
Expression of *lasR* regulon genes in clinical *lasR*^*WT*^, lasR* and lasR**/rhlR* isolates under biofilm growth conditions. Expression values (log_10_(nrpg + 1)) of **a** *rhlAB*, **b** *phzABCDEFG*, **c** *hcnABC*, **d** *aprIAFEDX*, **e** *pqsABCDE* in *lasR*^*WT*^ (*n* = 22), lasR* (*n* = 15), and lasR**/rhlR* (*n* = 7) clinical isolates under biofilm growth conditions are depicted. Asterisk: *p*-value < 0.05, Wilcoxon rank sum test or Welch’s *t*-test. Boxplot elements are: center line—median; box limits—upper and lower quartiles; whiskers—1.5× interquartile range; points—gene expression values (log_10_(nrpg + 1)). Reads are shown as log_10_ normalized million reads per gene.

Previous work has shown that RhlR is able to regulate the expression of *lasR*-dependent genes in late stationary growth conditions so that LasR becomes dispensable^29^. Here, we aimed at evaluating whether also under biofilm growth conditions, LasR becomes dispensable due to the activation of RhlR in a LasR independent manner. Therefore, we searched our clinical isolates for variants harboring inactivating mutations in both *lasR* as well as *rhlR*. We identified 22 isolates with no mutation in the two genes (WT), 15 isolates with an inactivating mutation (InDel/Stop) exclusively in *lasR* (lasR*), and 7 isolates with InDel/stop mutations in *rhlR* and an InDel/stop and/or non-synonymous mutations in *lasR* (lasR**/rhlR*). For all of the isolates both, planktonic and biofilm transcriptomes, were available. As expected, the isolates harboring a *rhlR* inactivating mutation exhibited a significantly reduced expression of *rhlI*, the autoinducer synthase, which is under the direct control of RhlR (Supplementary Fig. 4). We then analyzed the expression of genes that clearly belonged to the LasR core regulon (138 genes that were commonly positively regulated by LasR under planktonic conditions; Table 1) under biofilm conditions (Fig. 5). We found that the expression of *rhlAB*, *phzA-G*, and *hcnABC* was independent of a functional *lasR* gene under biofilm growth conditions. In the lasR* isolates, expression levels even exceeded those in the *lasR*^*WT*^ isolates. However, their expression was clearly dependent on a functional *rhlR* gene under biofilm growth conditions as the expression levels of these genes in the lasR**/rhlR* double mutants did not reach those of the wild-type. In accordance, the production of elastase was abolished in the majority of the lasR* and lasR**/rhlR* isolates when grown planktonically (shaking incubation for 24 h), whereas elastase production under biofilm conditions (static growth for 48 h) was at a similar level in lasR* but not in the lasR**/rhlR* strains as compared to *lasR* wild-type isolates (Supplementary Fig. 2c, d).

Our results thus demonstrate that RhlR acts downstream of LasR to impact the expression of many genes and that RhlR becomes activated under biofilm growth conditions independent of LasR to induce expression of genes that belong to the LasR core regulon under planktonic conditions. Supplementary Fig. 5 depicts the comparison of the expression of all LasR core regulon genes (*n* = 138) among the *lasR*^*WT*^, lasR* and lasR**/rhlR* isolates under planktonic and biofilm growth conditions. The data confirm that RhlR becomes activated under biofilm growth conditions independent of LasR to induce expression of the LasR-regulon genes.

Of note, the expression of the alkaline protease biosynthesis genes (*aprIAFEDX*) was dependent on *lasR* even under biofilm conditions and the additional inactivation of *rhlR* did not reduce gene expression further (Fig. 5d). In accordance, both, lasR* and lasR**/rhlR* mutant isolates showed in skim milk agar plate-grown colony biofilms a significantly reduced proteolytic activity as compared to *lasR*^*WT*^ strains (Supplementary Fig. 2b). Furthermore, the expression of the *Pseudomonas* Quinolone Signal (PQS) biosynthetic gene cluster (*pqsABCDE*) seemed to be independent of both *lasR* and *rhlR* under biofilm growth conditions (Fig. 5e).

**Fig. 6:**
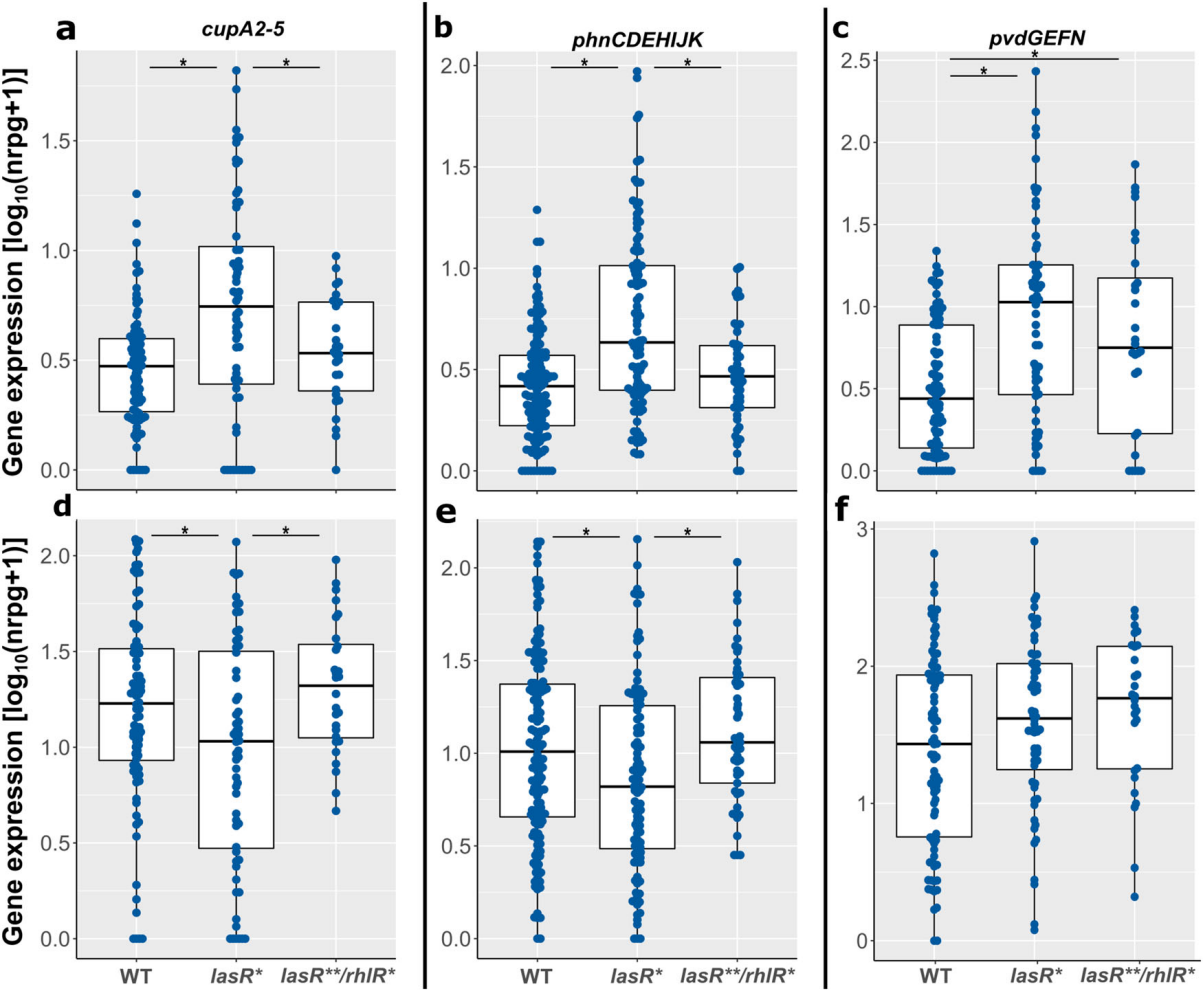
Expression of genes negatively affected by the presence of *lasR* in clinical *lasR*^*WT*^, lasR*, and lasR**/rhlR* isolates. Expression values are shown for planktonic (**a**–**c**) and biofilm growth conditions (**d**–**f**). Expression values (log_10_(nrpg + 1)) of (**a**, **d**) *cupA2-5*; (**b**, **e**) *phnCDEHIJK*; (**c**, **f**) *pvdGEFN* in *lasR*^*WT*^ (*n* = 22), lasR* (*n* = 15), and lasR**/rhlR* (*n* = 7) clinical isolates are depicted. Asterisk: *p*-value < 0.05, Wilcoxon rank sum test. Boxplot elements are: center line—median; box limits—upper and lower quartiles; whiskers—1.5× interquartile range; points—gene expression values (log_10_(nrpg + 1)). Reads are shown as log_10_ normalized million reads per gene.

We also analyzed the genes that were expressed at higher levels in the absence of *lasR* under planktonic growth conditions (Fig. 6). Again, we found that upregulation of genes in the *lasR* mutant under planktonic growth conditions (Fig. 6a–c) seemed to be dependent on a functional *rhlR* gene as the *cupA2-5, phnCDEHIJK* and *pvdGEFN* gene expression levels were back to the wild-type level in the double lasR**/rhlR* mutant isolates. Under biofilm growth conditions (Fig. 6d–f) these genes were not upregulated in the *lasR*-deficient isolates. Their expression level was even below that of the wild-type under biofilm conditions in the *lasR*-deficient isolates but back to normal in the lasR**/rhlR* clinical isolates. Again, as depicted in Supplementary Fig. 6, the comparison of the expression of all (*n* = 412) genes, which have been identified to be upregulated in the *lasR*-deficient isolates, among the *lasR*^*WT*^, lasR* and lasR**/rhlR* isolates confirm that RhlR acts downstream of LasR to induce the expression of genes in the absence of *lasR* under planktonic conditions. However, this *lasR*-mediated inhibition of gene expression is lost under biofilm conditions.

### *rhlR* and *phoB* dependent PA14 gene expression profiles under planktonic and biofilm growth conditions

**Fig. 7:**
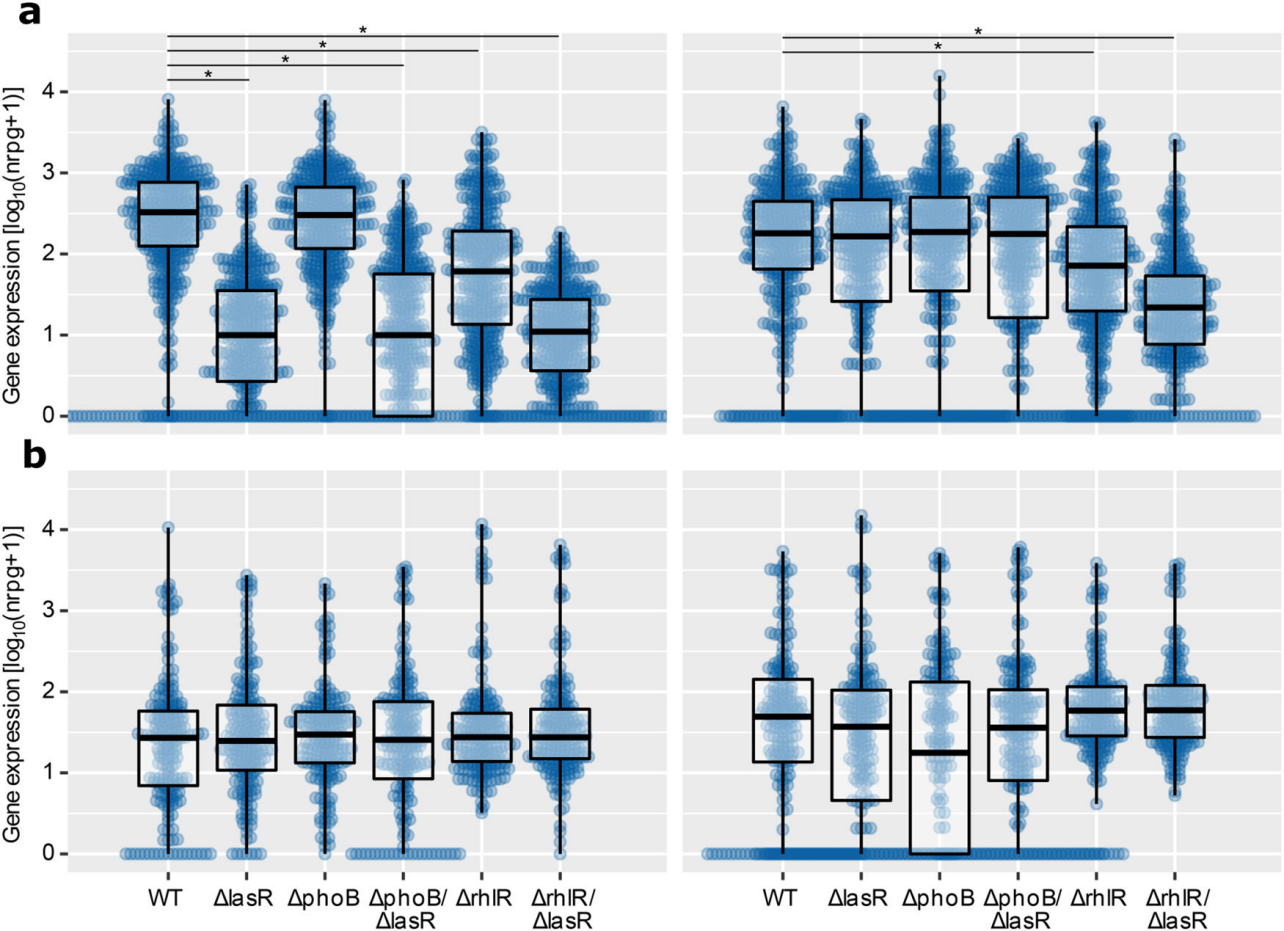
Expression of genes that are affected by the presence of *lasR* in the clinical lasR* isolates in PA14 and its isogenic ∆*lasR*, ∆*rhlR*, and ∆*phoB* single and double mutants. **a** Gene expression values (log_10_(nrpg + 1)) of the *lasR* core regulon genes (*n* = 136) and **b** of the *lasR* repressed (*n* = 412) genes in the ∆*lasR*, ∆*rhlR*, ∆*phoB*, ∆*phoB*/∆*lasR*, and ∆*lasR*/∆*rhlR* deletion mutants under planktonic (left) and biofilm (right) growth conditions. Asterisk: *p*-value < 0.05, Wilcoxon rank-sum test. Boxplot elements are: center line—median; box limits—upper and lower quartiles; whiskers—1.5x interquartile range; points—gene expression values (log_10_(nrpg + 1)).

We next evaluated whether *rhlR* acts downstream of *lasR* also in the PA14 type strain to induce the expression of genes in the absence of *lasR* under biofilm growth conditions. We, therefore, generated clean ∆*rhlR* and ∆*lasR/*∆*rhlR* PA14 deletion mutants using a CRISPR/Cas9-based approach and recorded their transcriptional profiles under planktonic and biofilm growth conditions. Again, we found that the expression of the *lasR* core regulon genes was dependent on the presence of *lasR* under planktonic, but not under biofilm conditions, while the lack of *rhlR* reduced the expression of the *lasR* regulon genes under both, planktonic and biofilm conditions (Fig. 7a). Since it has recently been demonstrated that LasR becomes dispensable under low phosphate growth conditions^30^, we also generated a deletion in the gene encoding the phosphate starvation response regulator PhoB as well as a ∆*lasR/*∆*phoB* mutant. However, the lack of *phoB* did not have any influence on the expression of the PA14 *lasR* core regulon genes (Fig. 7).

We also had a closer look at genes (*n* = 412) that were expressed at a higher level in the clinical lasR* vs. *lasR*^*WT*^ isolates. Expression of those genes was demonstrated to be less consistent across the clinical isolates (Fig. 3d). This indicates that the lack of *lasR* might not directly control their expression but rather that the clinical *lasR*-defective isolates tend to produce a certain, possibly compensatory, gene expression profile that can be repeatedly found in the clinical *lasR* mutants. If this is true, one would not necessarily expect to see this profile of gene expression in the clean PA14 *lasR* deletion mutant. Indeed, expression of the 412 genes that were upregulated in the clinical lasR* was not differentially regulated in either of the ∆*lasR*, ∆*rhlR*, and ∆*phoB* single and double mutants under planktonic or biofilm growth conditions (Fig. 7b).

## Discussion

In this study, we took advantage of the extensive transcriptome as well as whole-genome sequencing data on a large collection (>400) of clinical *P. aeruginosa* isolates^14,16^. Those strains have been isolated from different infected body sites and they were recovered from both, acute and chronic infections. In order to enable the successful growth of the highly diverse clinical isolates, we recorded the transcriptomes following growth under rich media (LB) conditions. Although growth e.g. in a synthetic sputum medium might have reflected the conditions within the human host more accurately, the non-selective cultivation conditions accommodated the growth of all clinical isolates. Despite the fact that the transcriptional profiles were recorded under rich medium growth conditions, the recorded transcriptional patterns are nevertheless the result of genomic variations that have evolved during the infection process in the human host.

The extensive data allowed us to cluster the clinical isolates according to a high vs. low expression status of a large subset of expressed genes. Although we did not account for absolute differences in gene expression levels, we were able to group isolates according to the complex high/low gene expression profiles that developed repeatedly in different *P. aeruginosa* strains. Our analysis identified mutations within *lasR* as major contributors to evolved gene expression patterns and therefore as main drivers of adaption towards the habitat of the human host. Indeed, inactivating mutations within *lasR* are well-known pathoadaptive mutations, frequently found in *P. aeruginosa* isolates from the respiratory tract of CF patients^23,31–33^. LasR-deficient isolates have been associated with advanced disease and major changes in the expression of QS-regulated genes^9,11,34^. However, especially the apparent reduced ability to produce virulence factors raises the question of the fitness advantage of such an evolutionary path^26,35,36^.

The analysis of engineered *lasR* deletion mutants in *P. aeruginosa* type strains has previously uncovered the LasR regulon^27,37^. This regulon comprises many virulence-associated genes, which are positively regulated by LasR activity. In this study, we identified genes that are commonly found to be differentially regulated in *lasR*-deficient (lasR*) versus *lasR*-proficient (*lasR*^*WT*^) clinical isolates. This core regulon included many genes, which were also found to be LasR-dependent in the PA14 type strain. However, we also found additional pathoadaptive traits in the lasR* isolates. Recording of transcriptional profiles of clinical lasR* isolates informed on important co-selected or possibly compensatory adaptations that might explain the success of the *lasR* mutations in the clinical context. Most remarkably, we found that the gene expression profile of the lasR* isolates did not differ to the same extent to those of *lasR*^*WT*^ clinical isolates when cultured under biofilm growth conditions. Indeed, it seems that the lasR* isolates exhibit a transcriptional profile typical for growth in a biofilm, already under planktonic culture conditions. The lasR* isolates were found to regulate 1.52-fold less genes compared to *lasR*^*WT*^ isolates (25% and 38% of the whole genome respectively, Supplementary Data 7) when switching to the biofilm growth mode. Genes encoding for the *cup* fimbriae^38^ and the exopolysaccharide Pel^39^ were already expressed under planktonic growth conditions in the lasR* isolates. Furthermore, despite the fact that the transcriptional profiles have been recorded under rich medium conditions, genes important for metabolizing complex carbon sources such as phosphatidylcholine or amino acids and for the assimilation of phosphate as well as iron uptake were highly expressed in the lasR* isolates. Amino acids and polyamines are major carbon sources in the CF lung^40,41^, while phosphate and iron are important for the establishment of an infection^42–44^.

In a process of genetic assimilation^45^, induced traits can lose their environmental sensitivity and thereby become robust to the environment (i.e. they no longer require the environmental signal for their expression). It seems that the switch to the biofilm mode of growth, which is expected to be triggered by the environmental conditions in the human host, might have become fixed in the lasR* population^45,46^.

The results of our study also showed that the genes of the *lasR* regulon do not depend on the presence of *lasR*, but on the presence of *rhlR* under biofilm growth conditions. An exception was the alkaline protease gene cluster (*apr*) and the PQS synthesis operon, whose expression remained dependent on the presence of a functional *lasR* even under biofilm growth conditions. It has been described previously that LasR becomes dispensable under conditions where RhlR is activated by different means, in order to drive genes belonging to the LasR regulon. A decoupling of the QS networks has been described under diverse environmental conditions such as low phosphate^30^, late stationary phase^28^ and now biofilm growth conditions. Interestingly, the phosphate-starvation regulator PhoB was previously shown to interact either directly or indirectly via RhlR and PqsR on the expression of the otherwise LasR-dependent pyocyanin synthesis gene clusters^47^. However, our results indicate that the phosphate starvation response regulator PhoB does not play a role in the activation of *lasR/rhlR* dependent genes under biofilm growth conditions, despite the fact that many genes that are activated in the clinical *lasR* mutants are involved in the acquisition of phosphate.

In conclusion, we demonstrate in this study that the analysis of large sample sizes of clinical isolates provides an interesting alternative to in vitro long-term experimental evolution experiments. Our data illustrate the power of profiling large sample sizes of clinical isolates to discover novel evolutionary trajectories in pathogen populations on their way of adaptation to the conditions encountered during an infection process within the human host. An integrated analysis of large-scale genomic and transcriptomic data together with clinical metadata, including detailed knowledge about the isolate-specific history (e.g. duration of infection and infection site), would further extend our knowledge and contribute to a more comprehensive understanding of adaptive evolution during infection. Profiling the consequences of pathoadaptive mutations promises to identify predictable pathways to successful long-term persistence within a host and thus highlights opportunities for developing novel strategies to combat therapy-refractory chronic infections.

## Methods

### Bacterial strains and growth conditions

Bacteria were grown in standard rich medium culture conditions (LB) with constant shaking (180 rpm) at 37 °C unless otherwise stated. All strains and plasmids used in this study are listed in Table 3. If necessary, LB medium was supplemented with gentamicin (Gm; 15 µg/ml for *E. coli* or 50 µg/ml for *P. aeruginosa*), or streptomycin (Sm; 50 µg/ml for *E. coli* or 500 µg/ml for *P. aeruginosa*) to retain plasmids.

**Table 3:** Strains and plasmids used in this study.

Strain	Genotype/description	Reference
*E. coli*		
DH5α	F-, *sup*E44, Δ*lac*U169, (ϕ80 *lac*ZDM15), *hsd*R17, (rk-mk^*+*^), *recA1*, *endA1*, *thi1*, *gyrA*, *relA*	^61^
*P. aeruginosa*		
PA14 WT	Wild type strain	^24^
PA14 ∆*lasR*	PA14 derivative with a full deletion of *lasR*	^36^
PA14 ∆*rhlR*	PA14 derivative with a full deletion of *rhlR*	This study
PA14 ∆*lasR* ∆*rhlR*	PA14 derivative carrying a double *lasR*/*rhlR* deletion	This study
PA14 ∆*phoB*	PA14 derivative with a full deletion of *phoB*	This study
PA14 ∆*lasR* ∆*phoB*	PA14 derivative carrying a double *lasR/phoB* deletion	This study
*Plasmids*		
pS448•CsR	Sm^R^, *oriV pRO1600/ColE1*, *oriT*, *xyl*S, *Pm*→*cas*9, *P*_*EM7*_→sgRNA	^48^
pSEVA658-*ssr*	Gm^R^, *oriV RSF1010*, *oriT*, *xyl*S, *Pm*→*ssr*	^62^
pSEVA624	Gm^R^, *oriV RK2*, *oriT*, *lac*I^q^/P_*trc*_ expression cassette	^63^
pSH624-*ssr*	Gm^R^, pSEVA624 cloned with the *ssr* recombinase sequence	This study
pS448•CsR-rhlR_spc2	Sm^R^, derivative of pS448•CsR carrying a *P*_*EM7*_→*rhlR*-targeting sgRNA	This study
pS448•CsR-phoB_spc2	Sm^R^, derivative of pS448•CsR carrying a *P*_*EM7*_→*phoB*-targeting sgRNA	This study

### Generation of scarless deletion mutants using a novel CRISPR/Cas9-recombineering method

We developed a targeted CRISPR/Cas9-recombineering system in order to generate clean deletion mutants in *P. aeruginosa*. For this endeavor, we resorted to the pS448·CsR plasmid developed for the accelerated genome engineering in the closely related bacterium species *P. putida*^48^. This vector controls the constitutive expression of a target-specific small guide RNA (sgRNA) and the inducible production of the Cas9 endonuclease upon addition of 3-methylbenzoate (3-*m*Bz). In *Pseudomonas* spp., it has been previously shown that using synthetic, linear DNA (either ssDNA or dsDNA) as a mutagenic template for genomic manipulations is futile unless an efficient DNA recombinase is also expressed^49,50^. Thus, we constructed a second vector expressing the gene encoding for the Ssr recombinase from *P. putida* DOT‐T1E^51^. The *ssr* coding sequence was excised from pSEVA658-*ssr* as an AvrII/BamHI restriction fragment and cloned anew into the same restriction sites of the isopropyl β-D-1-thiogalactopyranoside (IPTG)-inducible vector pSEVA624, resulting in plasmid pSH624-*ssr*.

For cloning each gene-specific spacer into pS448·CsR, we followed the indications by Wirth and collaborators^48^. Briefly, 100 pmol of each primer pair constituting the gene-specific spacer sequence were mixed and phosphorylated for 30 min at 37 °C in a 50-μl mixture with the T4 Polynucleotide Kinase (PNK, New England Biolabs). Next, the primers were denatured for 5 min at 95 °C and annealed to each other by allowing the mixture to slowly cool down to room temperature for at least 2 h. Both complementary oligonucleotides were designed to generate the protruding ends of the Eco31I restriction site (BsaI isoschizomer) when hybridizing (see Supplementary Table 1 for the details). Hence, it was possible to clone the dsDNA spacer fragment into the Eco31I restricted vector pS448·CsR (Table 3) to generate each gene-specific sgRNA-expressing vector. All oligonucleotides used for spacer construction, PCR amplification, as well as the 100 bp recombineering oligos for introducing the deletions are listed in Supplementary Table 1.

To achieve the deletions, *P. aeruginosa* PA14 or its ∆*lasR* derivative were previously electroporated with the recombineering plasmid pSH624-*ssr*. The *ssr*-carrying strains were grown overnight at 37 °C in 10 ml of LB medium supplemented with Gm. Next, 5 ml of fresh LB medium containing 50 µg/ml Gm and 1 mM IPTG were added to the cultures for inducing the *ssr* gene. After 3 h incubation at 37 °C, electrocompetent cells were prepared from 6 ml suspension of the induced cells as indicated by Choi and colleagues^52^, with the only modification that cells were finally resuspended in 200 μl of 0.3 M sucrose. Aliquots of 100 μl of cells were electroporated with 250 ng of the pS448•CsR-derivative harboring the appropriate spacer and 100 pmol of the mutagenic, recombineering oligonucleotide specific for each gene deletion (Supplementary Table 1). Cells were recovered for 2 hours in 2 ml of LB medium containing 2 mM 3-*m*Bz for the induction of the *cas9* gene. After incubation, *P. aeruginosa* cell dilutions were plated onto LB-Agar containing Gm, Sm and 2 mM 3-*m*Bz to select for plasmids and to counterselect the WT cells by means of CRISPR/Cas9 specific targeting of each spacer region. After 24 h of incubation at 37 °C, successfully generated deletion mutants were identified by colony PCR using primers flanking each gene (Supplementary Table 1). The efficiency of mutation was ~70% of the total colonies assayed. Altogether, this method allowed us to engineer *P. aeruginosa* mutants with clean deletions of *rhlR* and *phoB* within only 5 working days. Finally, the constructed mutant strains were cured of the plasmids by three consecutive passages in LB devoid of antibiotics and selected by sensitivity to the antibiotics and by PCR using primers specific for *ssr* and *cas9* genes (Supplementary Table 1).

### Transcriptional profiling of planktonic and biofilm-grown bacteria

We re-analyzed the transcriptional profiles of previously characterized clinical *P. aeruginosa* isolates collected across Europe^16,20^. Transcriptional profiles were recorded in LB, a universal, rich medium that allows the growth of a large variety of adapted clinical isolates. In the course of this study, we complemented the available dataset by recording transcriptomes of PA14 WT, as well as its deletion mutants (see Table 3) under identical planktonic^16^ and biofilm^14^ growth conditions. In brief, for planktonic growth 10 mL LB medium was inoculated with a starting optical density (OD_600_) of 0.05, and bacteria were cultivated at 37 °C and constant shaking (180 rpm) until they reached an OD_600_ of 2.02–2.28 (early stationary phase). For the harvest, 1 ml of bacterial suspension was mixed with an equal volume of RNAprotect (Qiagen), incubated for 10 min, and centrifuged at 8000 rpm for 10 min.

A randomly selected sub-group (*n* = 77) of our strain collection was chosen to be analyzed under biofilm growth conditions^14^. Biofilms were inoculated at a starting OD_600_ of 0.002 in LB and grown statically in half-area 96-well µClear microtiter plates (Greiner Bio-One) at 37 °C in a humid atmosphere. After 48 h, mature biofilms (10 wells per replicate) were resuspended and harvested in an equal volume of RNAlater (Qiagen). Three independent biological replicates were used for each strain and condition.

Total RNA from cell pellets was extracted using the RNeasy MiniKit (Qiagen) following an initial QIAshredder step according to the manufacturer’s instructions. DNA was removed by DNase I (Ambion) treatment, and the RNase inhibitor RNAsin (Promega) was added to protect the eluted RNA.

For cDNA library preparation^53,54^, total RNA was fragmented (150–350 bp) in Fast AP-Buffer (Thermo Scientific), DNA was digested by TURBO™ DNase (Invitrogen), and RNA fragments were phosphorylated by FastAP alkaline phosphatase (Thermo Scientific). Custom-made barcodes were ligated to the RNA using the T4 ligase (New England Biolabs) and fragments were subsequently purified and concentrated using the RNA Clean & Concentrator^™^-25 Kit (Zymo Research) following the manufacturer’s instructions. Ribosomal RNA was removed by using the RiboZero Bacteria kit (Illumina). cDNA libraries were synthesized using the SMARTScribe Reverse Transcriptase (Takara) followed by a PCR enrichment using the AccuPrime HiFi Taq polymerase (Invitrogen). Enzymatic reactions were carried out in the presence of SUPERase·In^™^ RNase Inhibitor (Invitrogen); RNACleanXP beads (Agencourt) were used for all RNA purification steps. Quality checks were performed before, during, and after cDNA library preparation with the RNA Nano Kit and an Agilent Bioanalyzer 2100 (Agilent Technologies). Libraries were sequenced on an Illumina HiSeq (single-end mode; 1 × 50 bp) or an Illumina NovaSeq 6000 (paired-end mode; 2 × 50 bp).

### Transcriptome analysis

Data analysis was performed in the *R* statistical environment (version 3.6.3)^55^ Reads were mapped against the UCBPP-PA14 reference genome (NCBI Reference sequence: NC_008463.1) using *stampy* (version 1.0.23)^56^. Normalization was performed using the command calcNormFactors() of the *R*-Package *edgeR* (version 3.28.1)^57^ and normalized read counts were extracted using the cpm() command of the same package. Genes with less than 1 count per million (cpm) in at least 21 isolates were excluded from the analysis. *p*-value correction outside of the *edgeR*-based differential gene expression analyses was calculated by p.adjust() of the *stats* R-package (version 3.6.1) with the adjustment method ‘fdr’.

Multimodality of gene expression was assessed by testing the normalized expression values of all genes of the tested 414 clinical *P. aeruginosa* isolates using the command modetest() of the *R*-package *multimode* (version 1.4)^58^ in default settings. Multimodality was assigned when the calculated and corrected *p*-value was <0.1. Isolates were sorted into either high or low expressing group by determining the last minimum before the last clear maximum of the distribution of expression values (see Supplementary Fig. 7). The expression of each gene that met the definition of a multimodal gene distribution was evaluated in each isolate and isolates were sorted into one of two groups accordingly: high or low expression of the respective gene.

Differential gene expression analysis was performed using the *R*-Package *edgeR* using the commands glmQLFTest() and topTags() with a corrected *p*-value of 0.05 as a cut-off for a significant differential gene expression and a threshold of log_2_FC ≥ 1 for upregulation and log_2_FC ≤ −1 for downregulation.

KEGG^24^ pathway enrichment was performed using the *KEGGREST* (version 1.26.1)^59^ package in *R* with the “pau” pathways to map the differentially regulated genes. Enrichment was performed via the p.hyper() function of the *stats* package with a corrected *p*-value of 0.05 as a cut-off for a significant enrichment.

The regulon robustness, as well as the core regulon analysis, were performed by calculating a differential gene expression of each individual lasR* isolates against all *lasR*^*WT*^ isolates (*n* = 28). The number of isolates with log_2_FC ≥ 1 or log_2_FC ≤ −1 for each gene was counted and summed over the number of tested lasR* isolates (*n* = 21). Finally, the *lasR* core regulon was determined as the intersection of genes that showed a log_2_FC above or below the respective threshold in at least 90% of the isolates (*n* = 19), and genes that are significantly regulated in the clean PA14 ∆*lasR* compared to PA14 WT.

MDS plots were calculated based on the normalized read counts created in the differential gene expression analysis described above and the PlotMDS() function in the *edgeR* package in *R*, taking all analyzed genes into account for the calculation of the dissimilarity matrix. The data was visualized using the *ggplot2 R*-package with the command stat_ellipse() for the 95% confidence interval ellipses.

Comparisons of gene cluster expression were generally performed by combining the complete transcriptional profiles of isolates of interest into a DGEList object as described above to subsequently perform the described normalization and low coverage gene exclusion. Expression values of the genes of interest were then extracted as library size-normalized counts per million (cpm() command in *R*; values described as “nrpg”) and a value of 1 was added to the all nrpg values prior to log_10_ transformation in order to prevent the creation of infinite values.

### DNA sequencing and SNP calling

In order to identify mutations in the sequences of *lasR* (PA14_45960) and *rhlR* (PA14_19120), previously published whole-genome sequencing data of 414 clinical isolates^16,17^ were screened for sequence variations in the respective genes. Mapping was accomplished using *stampy* (version 1.0.23)^56^ and variant calling was performed using *SAMtools* (version 0.1.19)^60^ with UCBPP-PA14 (NCBI Reference sequence: NC_008463.1) or PAO1 (NCBI Assembly: GCA_000006765.1 ASM676v1) as a reference. The strain background of the clinical isolates was assessed based on the phylogenetic analysis documented in Khaledi et al.^16^. PA7-like isolates were excluded in this study.

### Phenotypic characterization of clinical isolates

Phenotypic data for randomly selected clinical isolates representing a *lasR* wild-type allele status (*lasR*^*WT*^; *n* = 10), strains harboring a non-functional *lasR* (lasR*; *n* = 8) and isolates with inactivating mutations in both *lasR* and *rhlR* (lasR**/rhlR*; *n* = 4) was collected in previous studies^14,17^. In vivo virulence was determined 48 h post infection as % killing of *Galleria mellonella* larvae. For infection, bacterial strains were grown overnight and serially diluted in PBS. 20 µl of the bacterial suspension was injected into the last left proleg of the larvae with an MOI (multiplicity of infection) of 100 cfu (10 larvae per bacterial strain). Relative proteolytic activity was assessed by measuring clearing zones of spotted bacteria on cation-adjusted Müller-Hinton (MH) broth containing 10% (v/v) milk after 24 h of incubation. Overnight cultures were adjusted to an OD_600_ of 0.025 and 5 μl of the bacterial suspensions were spotted on top of the agar plates. An Elastin Congo Red (ECR) assay was applied to determine the elastolytic activity of secreted proteases. Bacteria were grown for 24 h in planktonic cultures (shaking at 180 rpm), or for 48 h in biofilms (static incubation) as described above. After harvest, 100 µl of bacteria-free culture broth was incubated with the substrate (900 μl ECR buffer containing 100 mM Tris [pH 7.5], 1 mM CaCl_2_, supplemented with 22.5 mg/ml ECR [Sigma-Aldrich]) for 3 h at 37 °C and 900 rpm. Elastase secretion was determined by measuring the absorbance of the supernatant at OD = 495 nm.

### Statistical analysis

The shapiro.test() function of the *stats* package in *R* (v 3.6.1) was applied to test for normal distribution of the compared groups. If no normal distribution was observed (*p*-value < 0.05) in one of the groups, the wilcox.test() command with default settings from the same package was employed to test for statistical significance.

Overrepresentation analysis was calculated using the phyper() command of the *stats* package in *R* with the *lower.tail* option set to “FALSE” to test for enrichment.

Statistical analyses for phenotypic data were performed in GraphPad Prism (v 8.3.0) by using Kruskal-Wallis (comparison of three groups) and Mann–Whitney tests (pair-wise comparison).

### Reporting summary

Further information on research design is available in the Nature Research Reporting Summary linked to this article.

### Supplementary information

Supplementary Information

Supplementary Data 1

Supplementary Data 2

Supplementary Data 3

Supplementary Data 4

Supplementary Data 5

Supplementary Data 6

Supplementary Data 7

Reporting Summary

## Data Availability

RNA- and DNA-sequencing data (clinical isolates) that support the findings of this study have been recorded previously^14,16,17,20^ and deposited in Omnibus GSE123544, Omnibus GSE134231, and the Sequence Read Archive PRJNA526797. RNA-sequencing data obtained for PA14 WT and the respective mutants (recorded in planktonic and biofilm growth conditions) are deposited in Omnibus GSE191103.

## Code availability

Scripts for RNA-seq data processing and analysis are available from the authors upon request.
