# Supplementary material for: Evolution of biofilm-adapted gene expression profiles in lasR-deficient clinical Pseudomonas aeruginosa isolates
Source: NPJ Biofilms Microbiomes. 2022 Feb 14;8:6. doi: 10.1038/s41522-022-00268-1 (PMC8844440; doi:10.1038/s41522-022-00268-1)
Supplement: Supplementary file 1 — Supplementary Information [file 41522_2022_268_MOESM1_ESM.pdf]

Supplementary Information

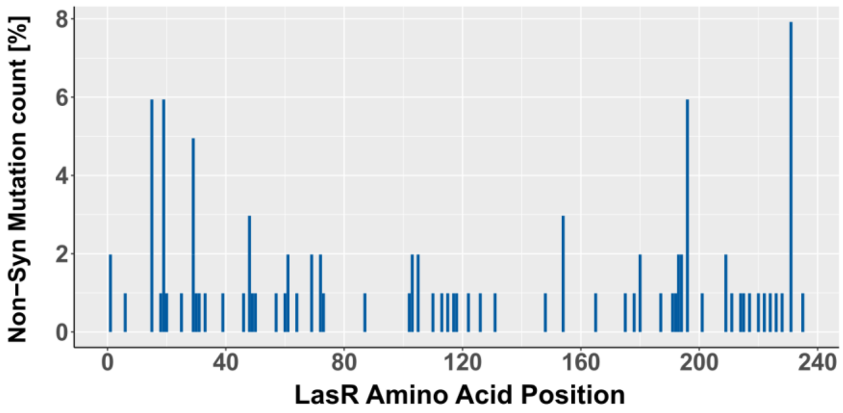

Supplementary Figure 1: Mapping of the non-synonymous amino acid exchanges to the LasR PAO1 and PA14 reference. The *lasR* gene sequences of all 414 clinical isolates that harbored non-synonymous sequence variations within *lasR* were mapped to their amino acid position within the protein. The *lasR* gene sequence of PA14 and PAO1 served as a reference. The relative number of clinical isolates harboring the respective amino acid variation is shown. Insertions/Deletions and premature stop codons were excluded.

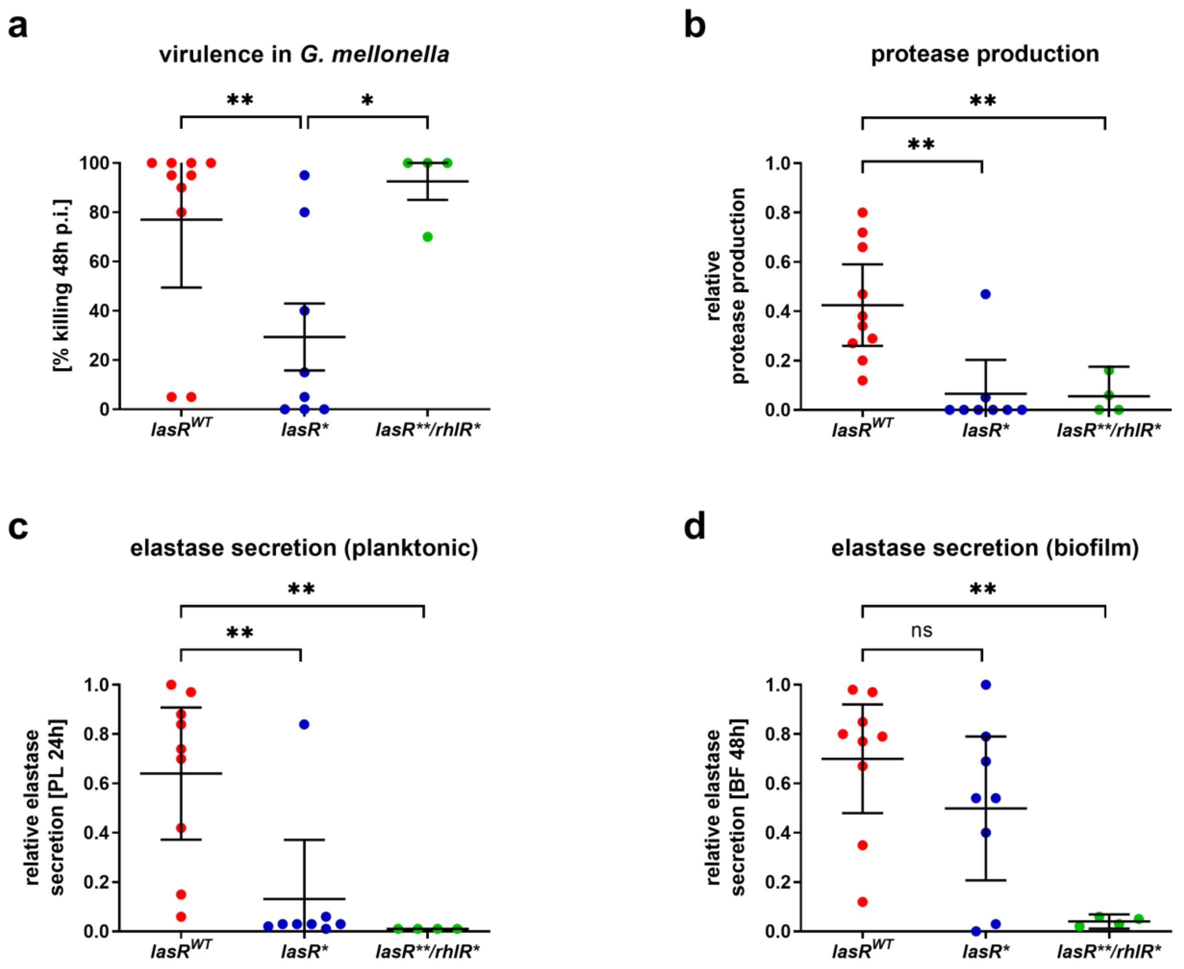

Supplementary Figure 2: Selected clinical isolates exhibit distinct phenotypic characteristics based on their allele status of the *lasR* and *rhlR* genes. Strains with a defective LasR (*lasR**) are significantly less virulent than wild-type isolates (*lasR*^{WT}) or strains lacking functional LasR and RhlR (*lasR***/*rhlR**) in an *in vivo Galleria mellonella* infection model (**a**). Protease production and elastase secretion (planktonic growth) are significantly reduced in *lasR** and *lasR***/*rhlR** isolates compared with *lasR*^{WT} (**b** + **c**). Elastase secretion for *lasR**, but not *lasR***/*rhlR** strains, is restored when cultivated under biofilm growth conditions (**d**). Each dot represents an individual clinical isolate. The solid line represents the mean and the whiskers display the 95 %-confidence intervals. Levels of statistical significance were calculated using Kruskal-Wallis and Mann-Whitney tests and are indicated as ** (p-value < 0.01), or * (p-value < 0.05).

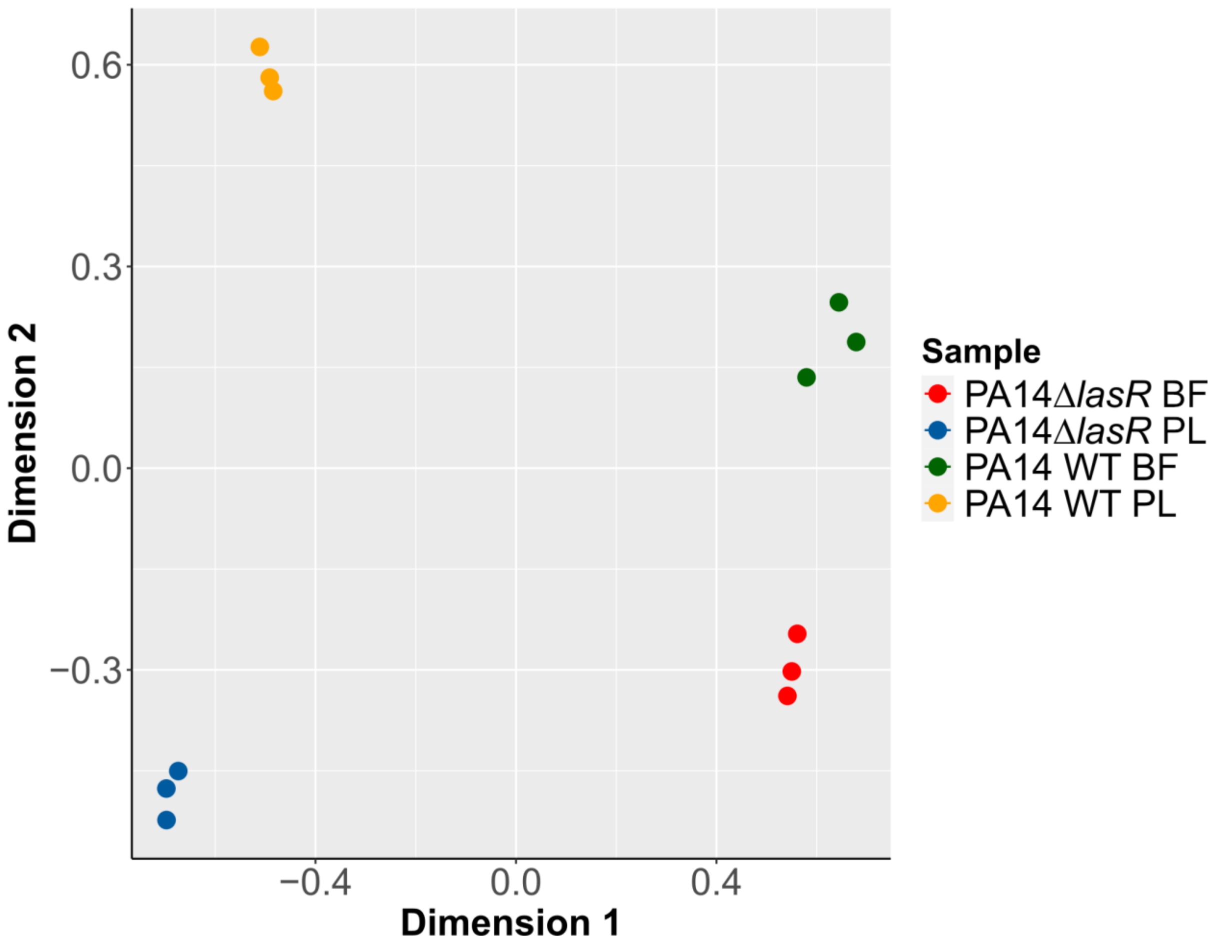

Supplementary Figure 3: Transcriptomes of *P. aeruginosa* PA14 and its Δ*lasR* mutant under biofilm and planktonic growth conditions. MDS plot depicts normalized global gene expression values of PA14 WT and its clean *lasR* deletion mutant (PA14Δ*lasR*) under planktonic growth conditions (in yellow and blue) and 48h biofilm growth (in green and red).

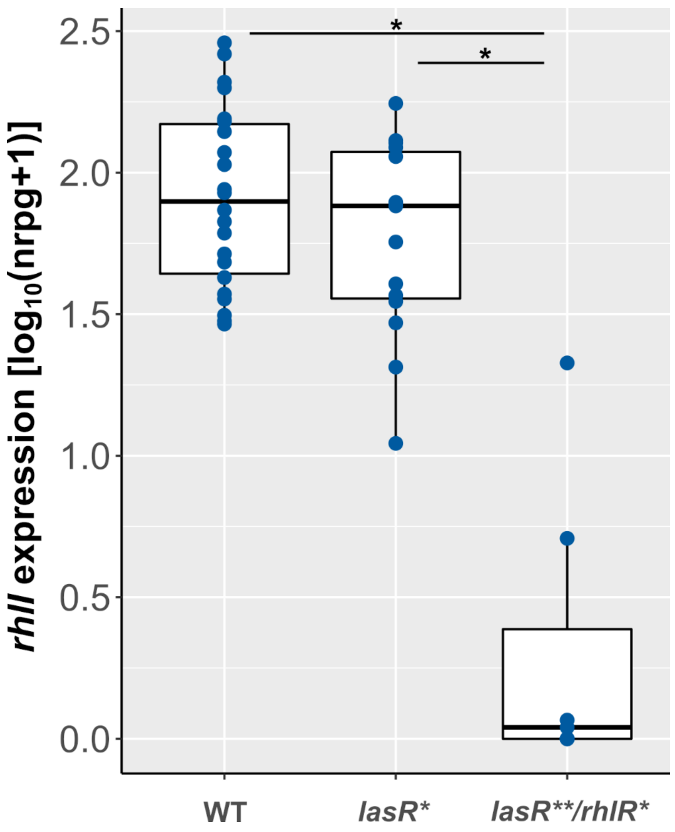

Supplementary Figure 4: *lasR/rhlR** clinical isolates exhibit a reduced *rhlI* expression compared to *lasR** and wild-type isolates.** Expression of *rhlI* encoding the autoinducer synthase RhlI in clinical *P. aeruginosa* isolates is depicted. Clinical isolates with InDel mutations or premature stop codons in either only *lasR* (*lasR**; n = 15), both *lasR* and *rhlR* (*lasR**/rhlR**; n = 7) or in none of the two genes (WT; n = 22) were included in the analysis. The 7 *lasR**/rhlR** isolates also included strains exhibiting non-synonymous amino acid exchanges in the gene sequence of *lasR*. Reads are shown as $\log_{10}$ normalized million reads per gene. A value of 1 was added to all nrpg values prior to $\log_{10}$ transformation in order to prevent the creation of infinite values. Each box includes 50% of the data. *p*-values < 0.05 (Wilcoxon rank sum test) are marked with an asterisk.

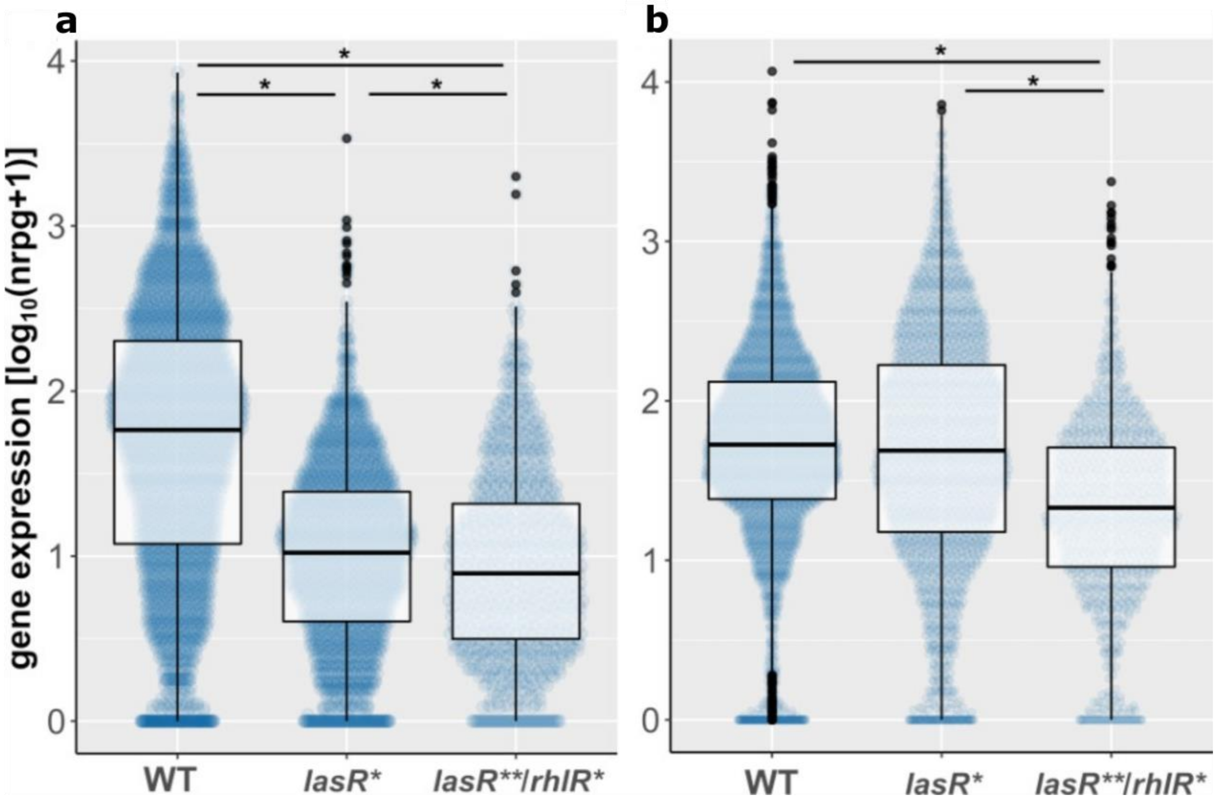

Supplementary Figure 5: Expression of all LasR core regulon genes (n = 138) among the *lasR*^{WT}, *lasR and *lasR**/rhlR** isolates.** Gene expression values ($\log_{10}$(nrpg+1)) of the core *lasR* regulon genes in the *lasR*^{WT} (n = 22), *lasR** (n = 15) and *lasR**/rhlR** (n = 7) clinical isolates under planktonic (**a**) and biofilm (**b**) growth conditions. Asterisk: p-value < 0.05 (Wilcoxon rank sum test).

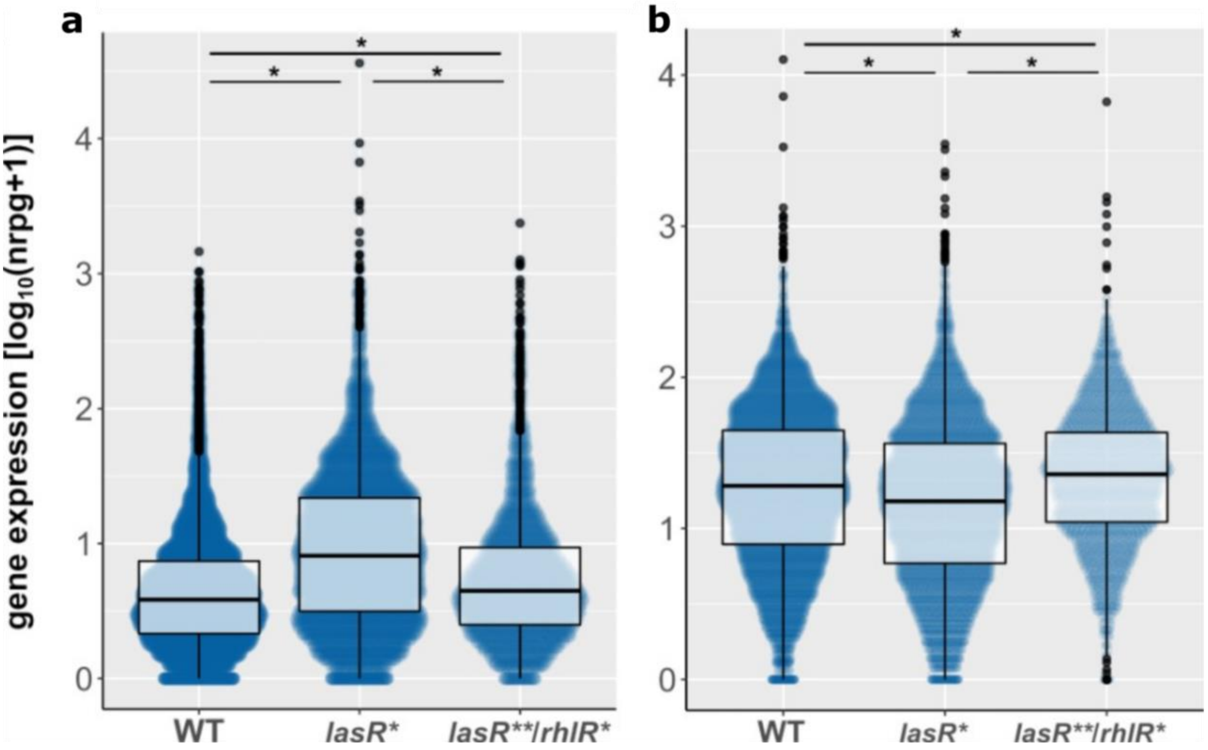

Supplementary Figure 6: Expression of all genes that are negatively affected by the presence of *lasR* among the *lasR*^{WT}, *lasR and *lasR***/*rhlR** isolates.** Gene expression values ($\log_{10}$(nrpg+1)) of the *lasR* repressed genes in the *lasR*^{WT} (n = 22), *lasR** (n = 15) and *lasR***/*rhlR** (n = 7) clinical isolates under planktonic (**a**) and biofilm (**b**) growth conditions. Asterisk: *p*-value < 0.05 (Wilcoxon rank sum test).

Supplementary Figure 7: Bi-modal genes show characteristic expression patterns in a collection of 414 clinical *P. aeruginosa* isolates. Figures display distributions and cut-off visualization. Density plots for each of the identified 136 genes with an at least bi-modal pattern display the expression distribution (based on normalized expression values; y-axis) across the population of strains (x-axis). Multimodality was calculated using the R package *multimode* in default mode, a corrected *p*-value of < 0.1 was used as a threshold. Red lines indicate expression cut-offs between lowly expressing and highly expressing isolates. n = number of analyzed isolates; Bandwidth = value used in the density() function (default mode) of R to smoothen the plot.

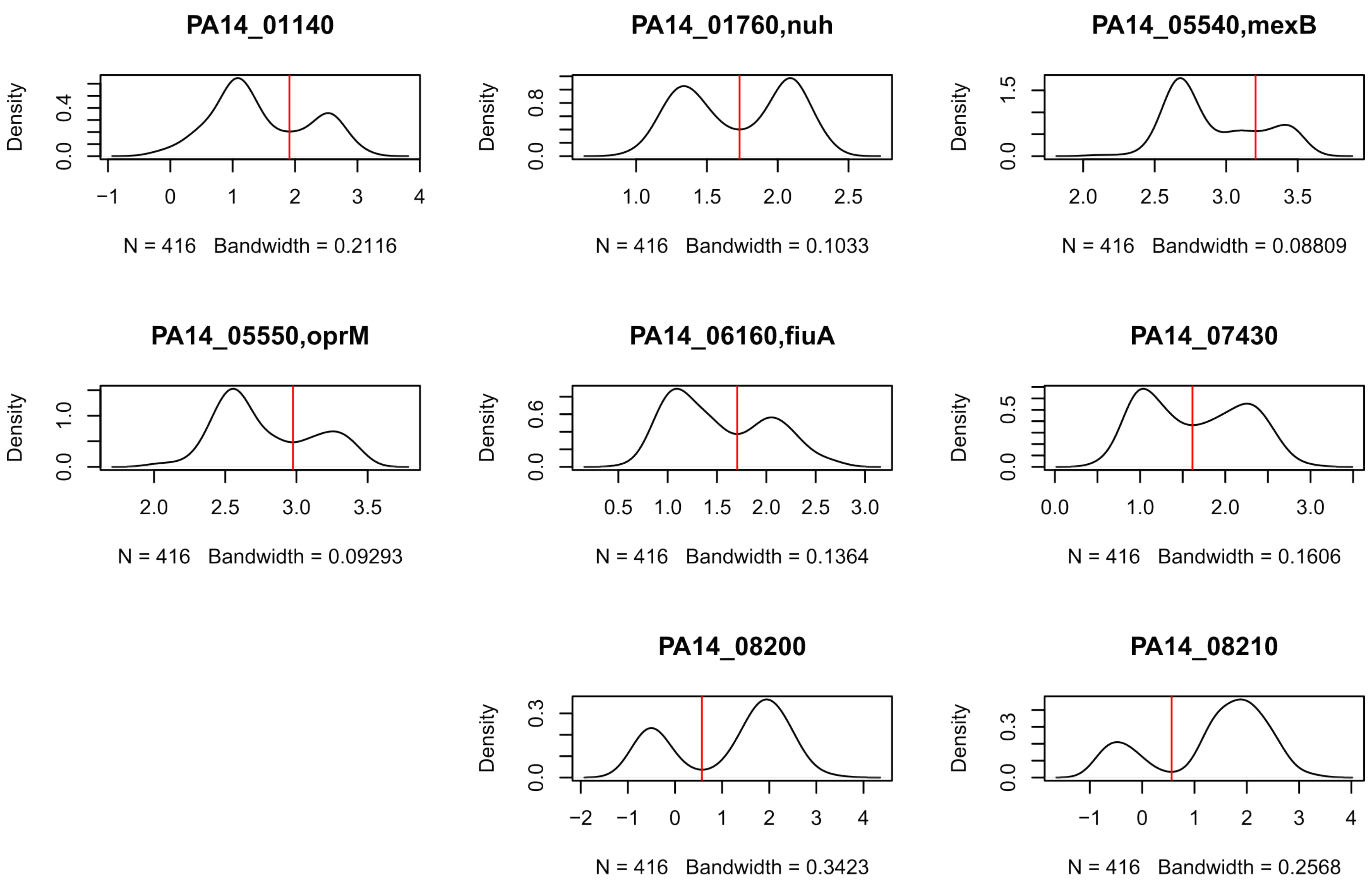
PA14_01140
Density
N = 416 Bandwidth = 0.2116
PA14_01760,nuh
Density
N = 416 Bandwidth = 0.1033
PA14_05540,mexB
Density
N = 416 Bandwidth = 0.08809
PA14_05550,oprM
Density
N = 416 Bandwidth = 0.09293
PA14_06160,fiuA
Density
N = 416 Bandwidth = 0.1364
PA14_07430
Density
N = 416 Bandwidth = 0.1606
PA14_08200
Density
N = 416 Bandwidth = 0.3423
PA14_08210
Density
N = 416 Bandwidth = 0.2568

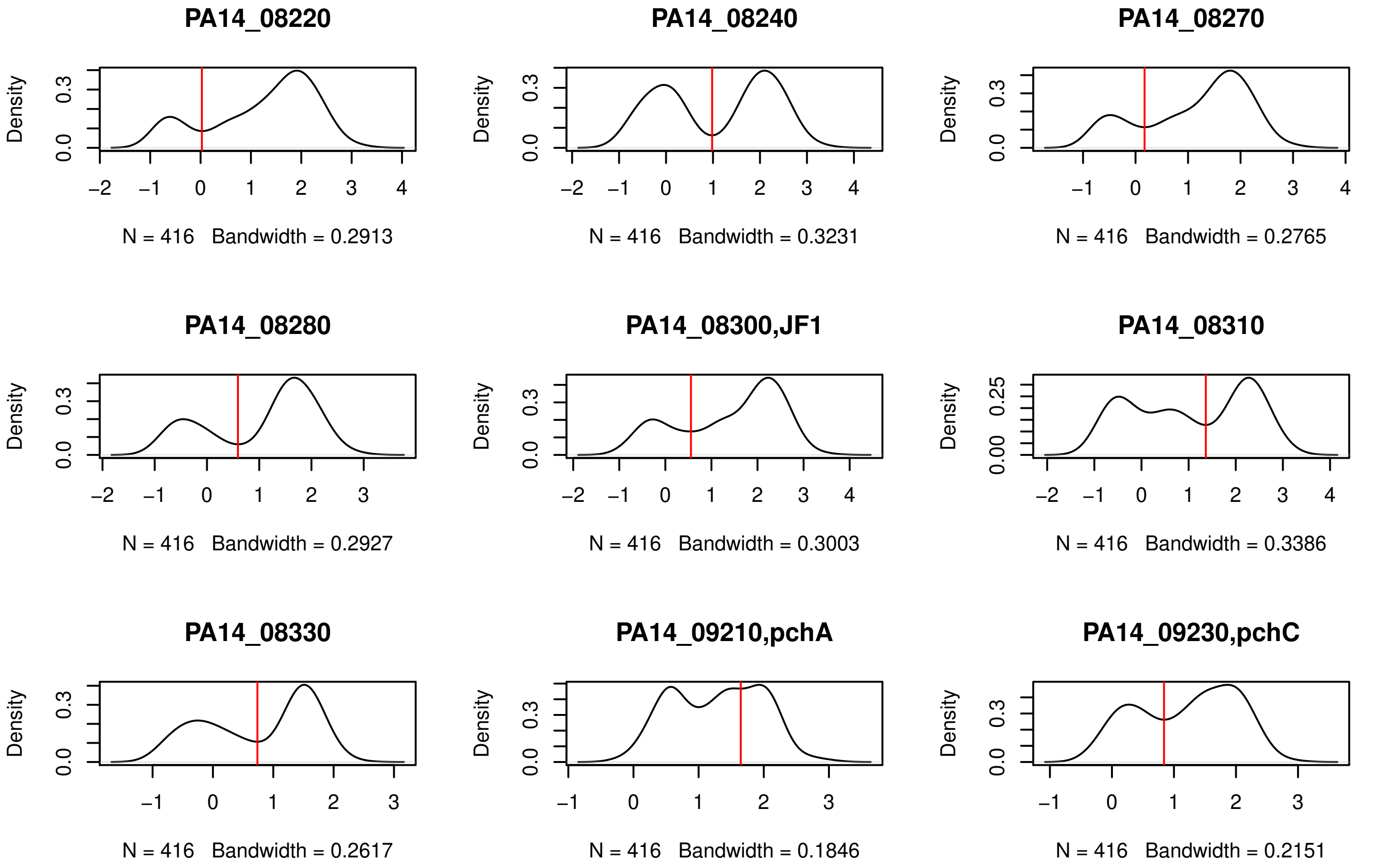
PA14_08220
Density
0.0
0.3
−2
−1
0
1
2
3
4
N = 416 Bandwidth = 0.2913
PA14_08240
Density
0.0
0.3
−2
−1
0
1
2
3
4
N = 416 Bandwidth = 0.3231
PA14_08270
Density
0.0
0.3
−1
0
1
2
3
4
N = 416 Bandwidth = 0.2765
PA14_08280
Density
0.0
0.3
−2
−1
0
1
2
3
N = 416 Bandwidth = 0.2927
PA14_08300,JF1
Density
0.0
0.3
−2
−1
0
1
2
3
4
N = 416 Bandwidth = 0.3003
PA14_08310
Density
0.00
0.25
−2
−1
0
1
2
3
4
N = 416 Bandwidth = 0.3386
PA14_08330
Density
0.0
0.3
−1
0
1
2
3
N = 416 Bandwidth = 0.2617
PA14_09210,pchA
Density
0.0
0.3
−1
0
1
2
3
N = 416 Bandwidth = 0.1846
PA14_09230,pchC
Density
0.0
0.3
−1
0
1
2
3
N = 416 Bandwidth = 0.2151

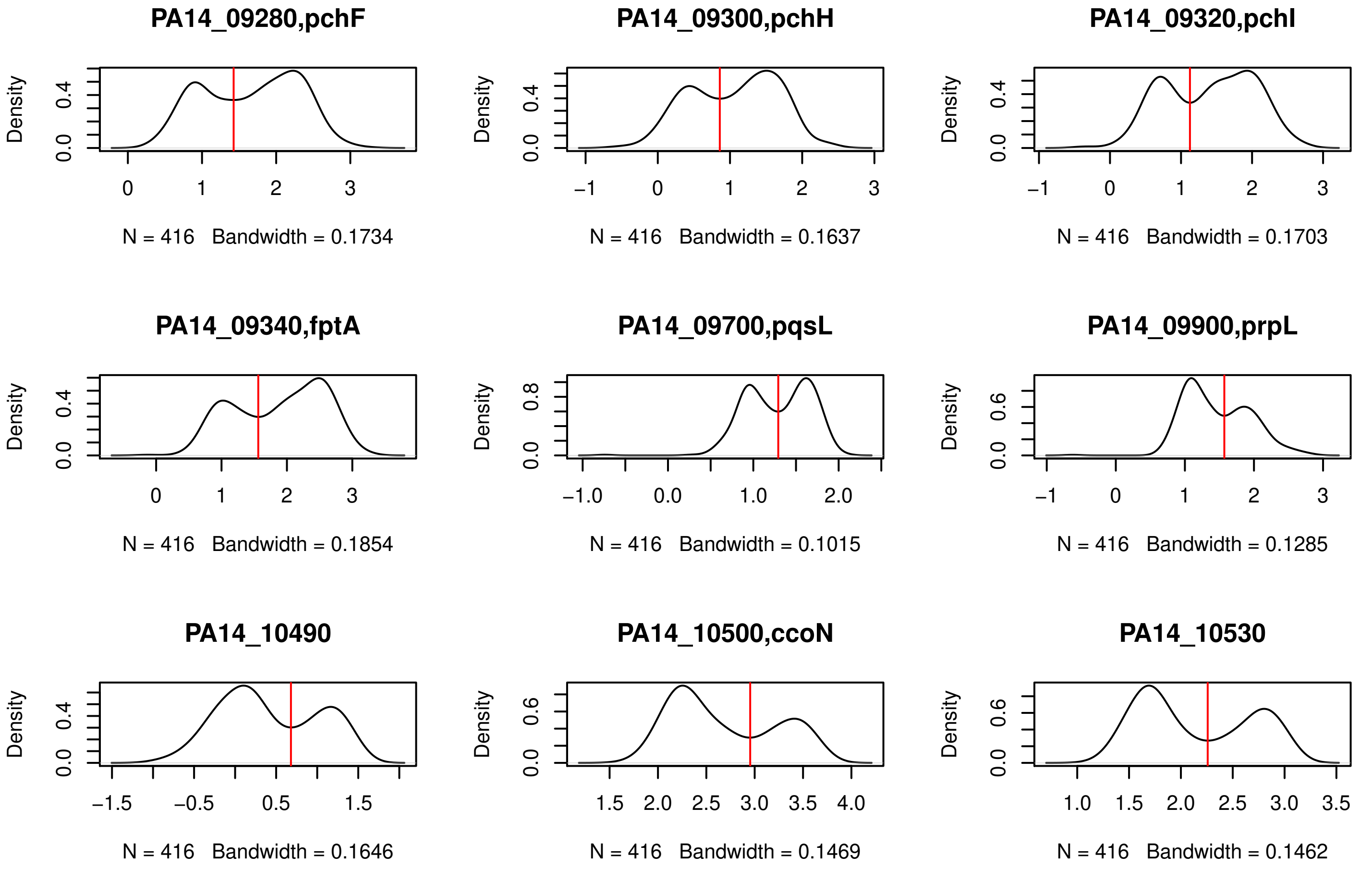

PA14_09280,pchF
Density
N = 416 Bandwidth = 0.1734
PA14_09300,pchH
Density
N = 416 Bandwidth = 0.1637
PA14_09320,pchI
Density
N = 416 Bandwidth = 0.1703
PA14_09340,fptA
Density
N = 416 Bandwidth = 0.1854
PA14_09700,pqsL
Density
N = 416 Bandwidth = 0.1015
PA14_09900,prpL
Density
N = 416 Bandwidth = 0.1285
PA14_10490
Density
N = 416 Bandwidth = 0.1646
PA14_10500,ccoN
Density
N = 416 Bandwidth = 0.1469
PA14_10530
Density
N = 416 Bandwidth = 0.1462

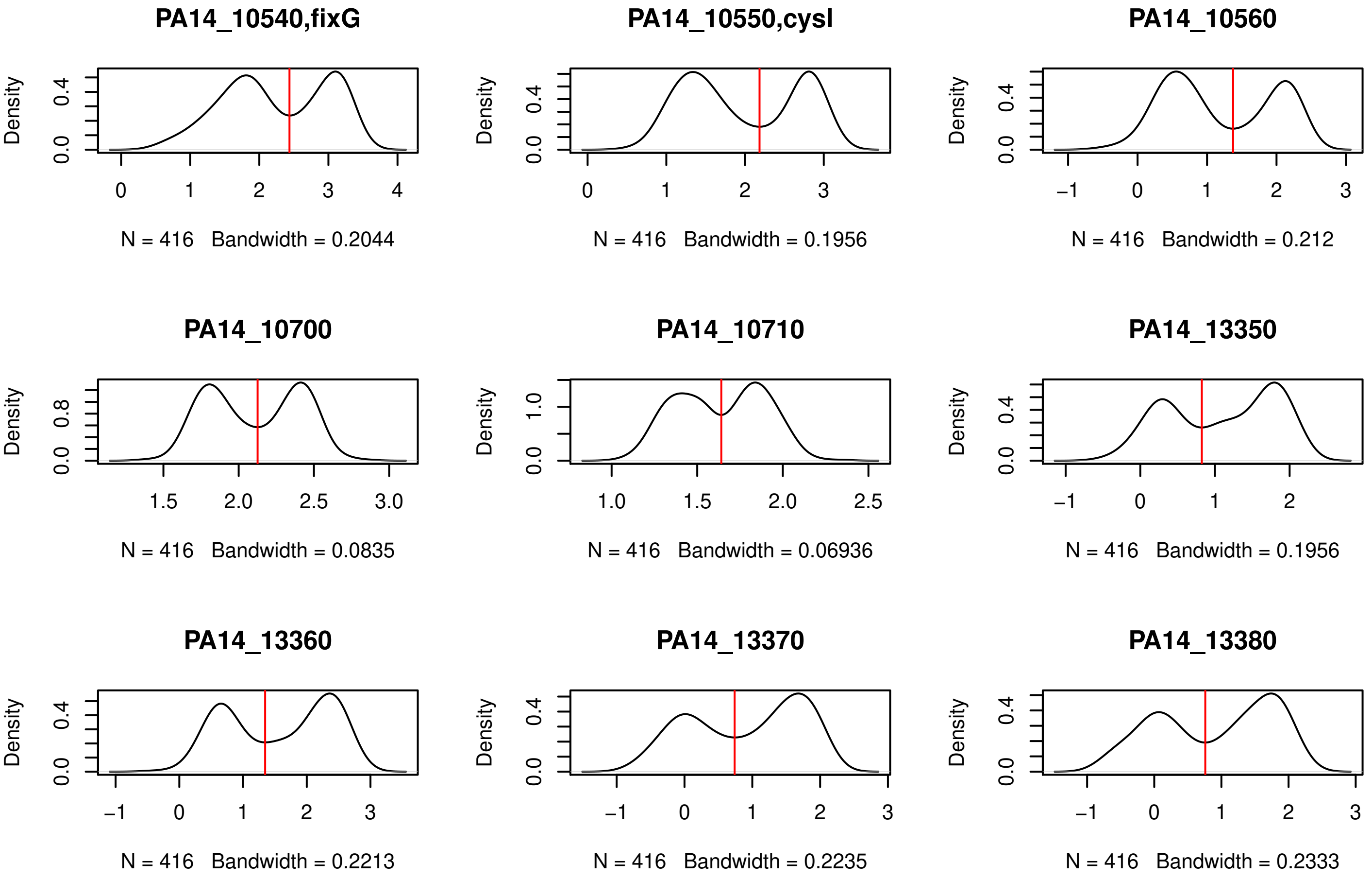
PA14_10540,fixG
Density
N = 416 Bandwidth = 0.2044
PA14_10550,cysI
Density
N = 416 Bandwidth = 0.1956
PA14_10560
Density
N = 416 Bandwidth = 0.212
PA14_10700
Density
N = 416 Bandwidth = 0.0835
PA14_10710
Density
N = 416 Bandwidth = 0.06936
PA14_13350
Density
N = 416 Bandwidth = 0.1956
PA14_13360
Density
N = 416 Bandwidth = 0.2213
PA14_13370
Density
N = 416 Bandwidth = 0.2235
PA14_13380
Density
N = 416 Bandwidth = 0.2333

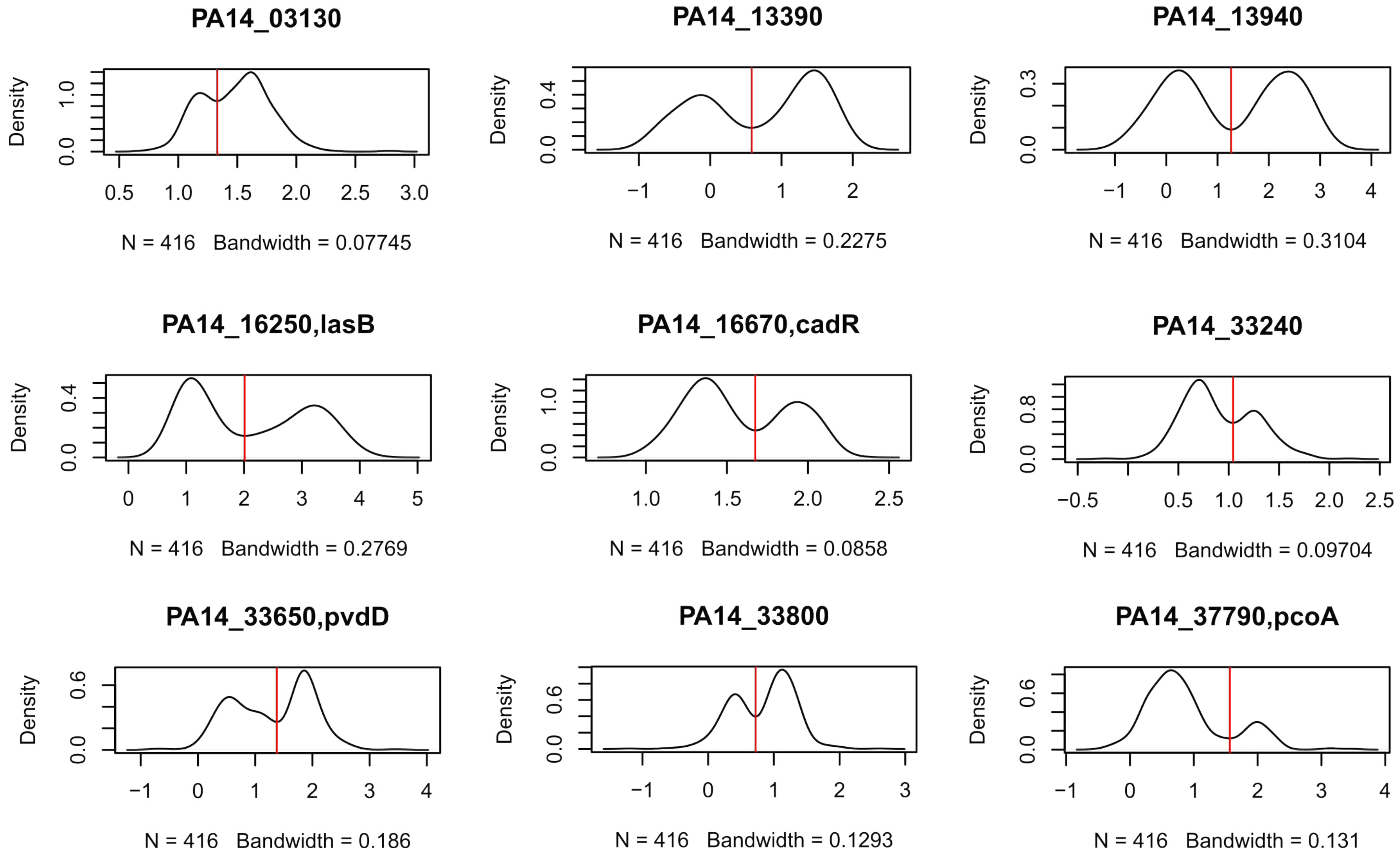
PA14_03130
Density
0.0
1.0
0.5
1.0
1.5
2.0
2.5
3.0
N = 416 Bandwidth = 0.07745
PA14_13390
Density
0.0
0.4
−1
0
1
2
N = 416 Bandwidth = 0.2275
PA14_13940
Density
0.0
0.3
−1
0
1
2
3
4
N = 416 Bandwidth = 0.3104
PA14_16250,lasB
Density
0.0
0.4
0
1
2
3
4
5
N = 416 Bandwidth = 0.2769
PA14_16670,cadR
Density
0.0
1.0
1.0
1.5
2.0
2.5
N = 416 Bandwidth = 0.0858
PA14_33240
Density
0.0
0.8
−0.5
0.5
1.0
1.5
2.0
2.5
N = 416 Bandwidth = 0.09704
PA14_33650,pvdD
Density
0.0
0.6
−1
0
1
2
3
4
N = 416 Bandwidth = 0.186
PA14_33800
Density
0.0
0.6
−1
0
1
2
3
N = 416 Bandwidth = 0.1293
PA14_37790,pcoA
Density
0.0
0.6
−1
0
1
2
3
4
N = 416 Bandwidth = 0.131

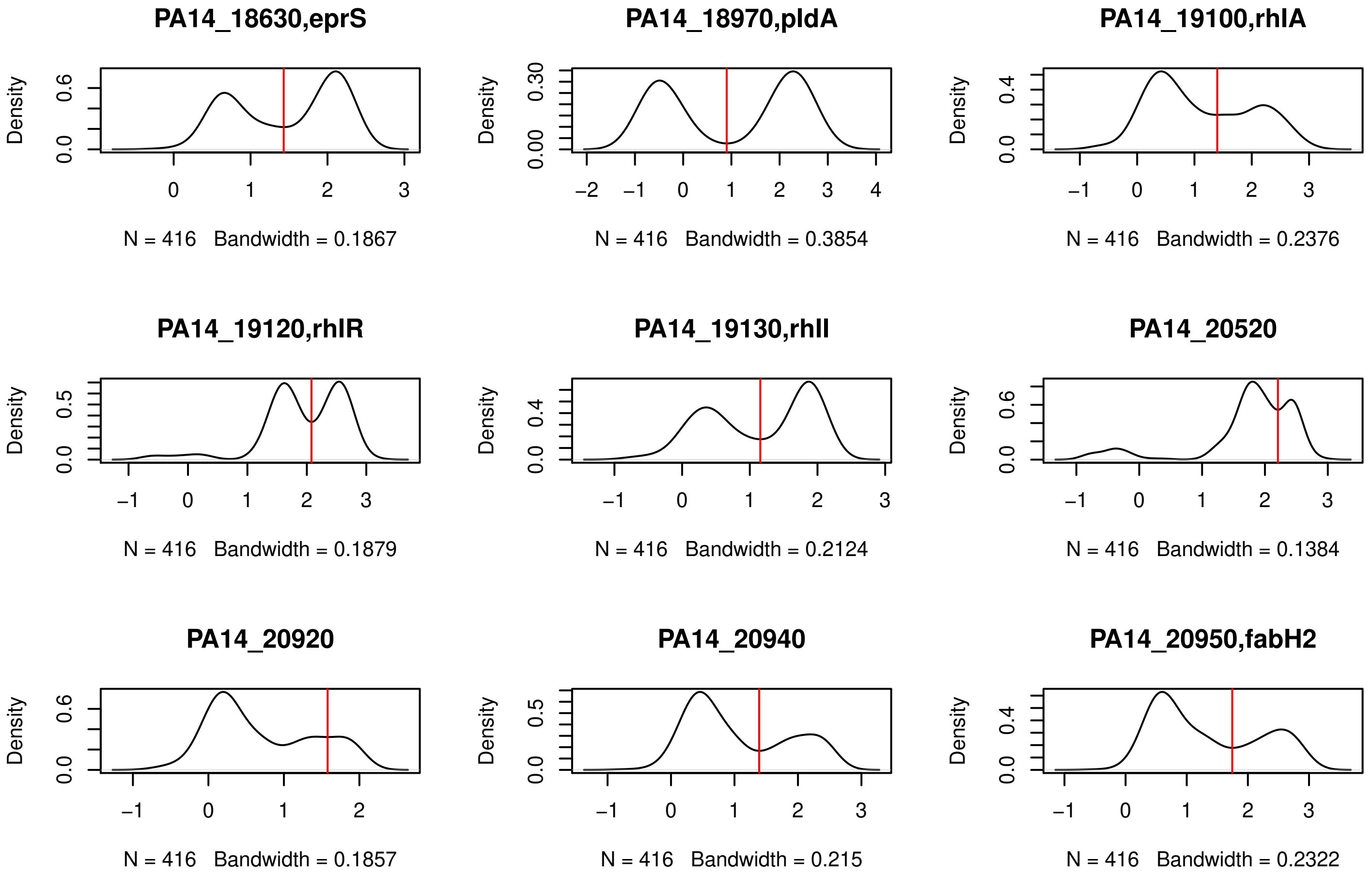
PA14_18630,eprS
Density
N = 416 Bandwidth = 0.1867
PA14_18970,pldA
Density
N = 416 Bandwidth = 0.3854
PA14_19100,rhlA
Density
N = 416 Bandwidth = 0.2376
PA14_19120,rhlR
Density
N = 416 Bandwidth = 0.1879
PA14_19130,rhlI
Density
N = 416 Bandwidth = 0.2124
PA14_20520
Density
N = 416 Bandwidth = 0.1384
PA14_20920
Density
N = 416 Bandwidth = 0.1857
PA14_20940
Density
N = 416 Bandwidth = 0.215
PA14_20950,fabH2
Density
N = 416 Bandwidth = 0.2322

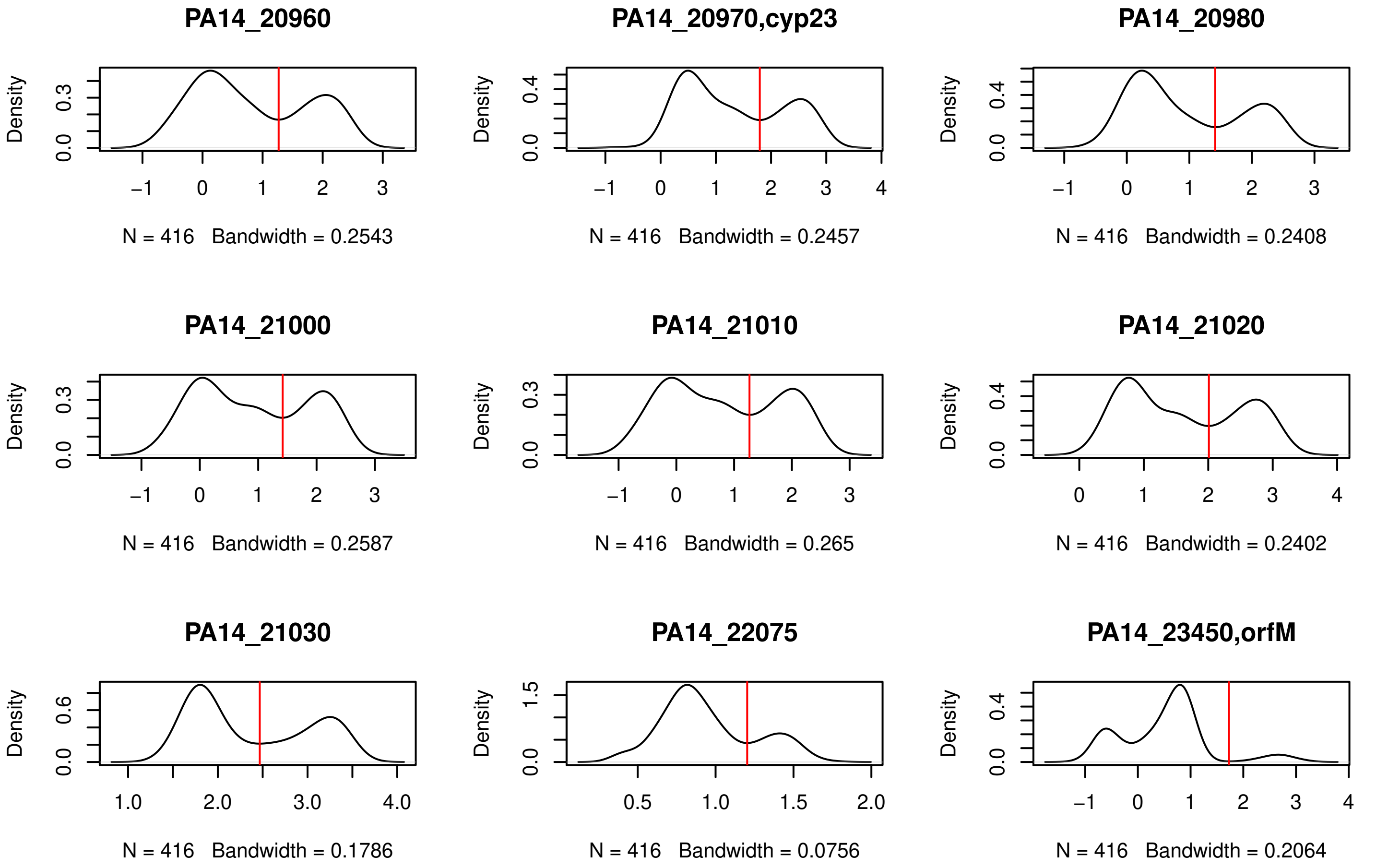
PA14_20960
Density
N = 416 Bandwidth = 0.2543
PA14_20970,cyp23
Density
N = 416 Bandwidth = 0.2457
PA14_20980
Density
N = 416 Bandwidth = 0.2408
PA14_21000
Density
N = 416 Bandwidth = 0.2587
PA14_21010
Density
N = 416 Bandwidth = 0.265
PA14_21020
Density
N = 416 Bandwidth = 0.2402
PA14_21030
Density
N = 416 Bandwidth = 0.1786
PA14_22075
Density
N = 416 Bandwidth = 0.0756
PA14_23450,orfM
Density
N = 416 Bandwidth = 0.2064

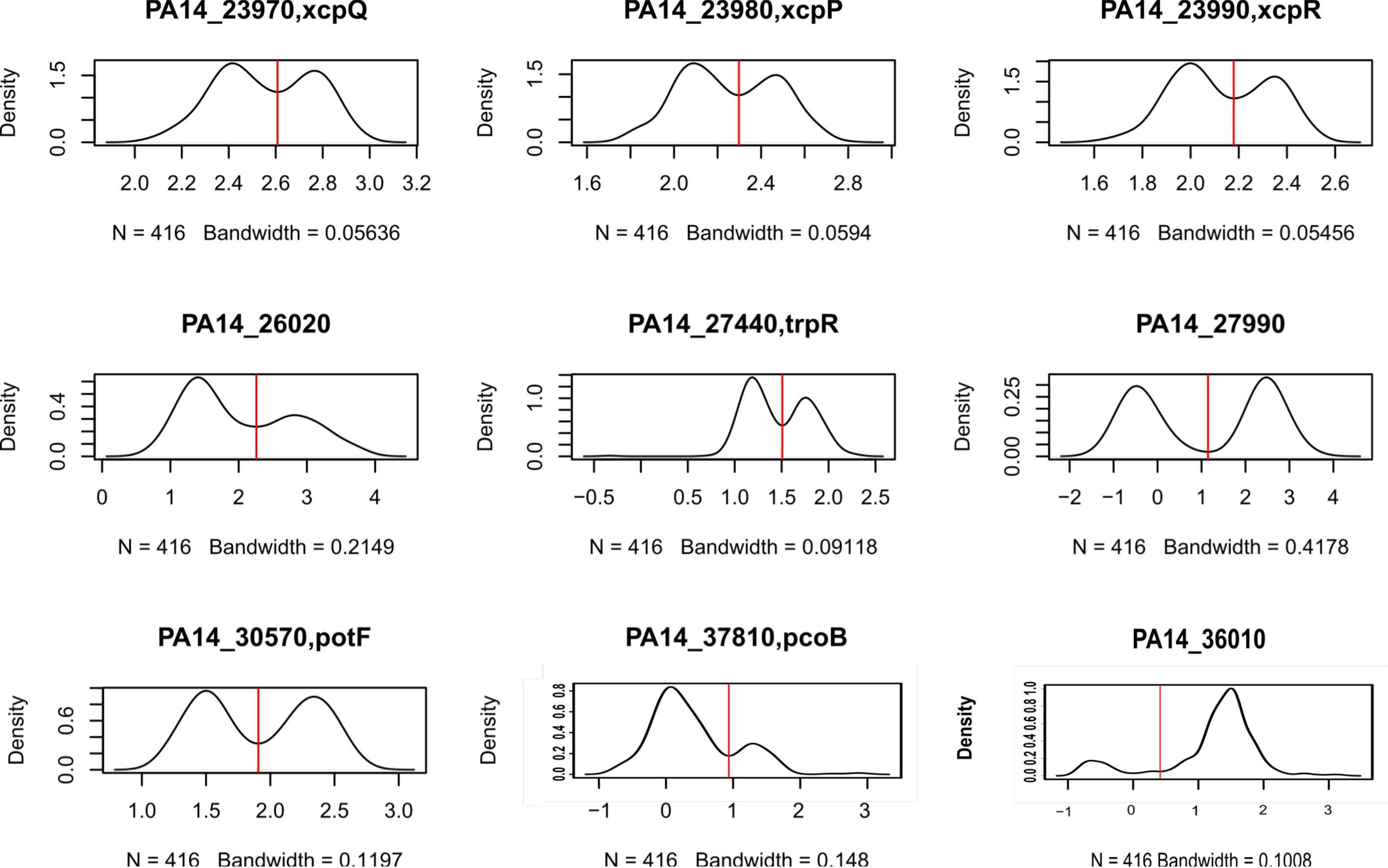
PA14_23970,xcpQ
Density
N = 416 Bandwidth = 0.05636
PA14_23980,xcpP
Density
N = 416 Bandwidth = 0.0594
PA14_23990,xcpR
Density
N = 416 Bandwidth = 0.05456
PA14_26020
Density
N = 416 Bandwidth = 0.2149
PA14_27440,trpR
Density
N = 416 Bandwidth = 0.09118
PA14_27990
Density
N = 416 Bandwidth = 0.4178
PA14_30570,potF
Density
N = 416 Bandwidth = 0.1197
PA14_37810,pcoB
Density
N = 416 Bandwidth = 0.148
PA14_36010
Density
N = 416 Bandwidth = 0.1008

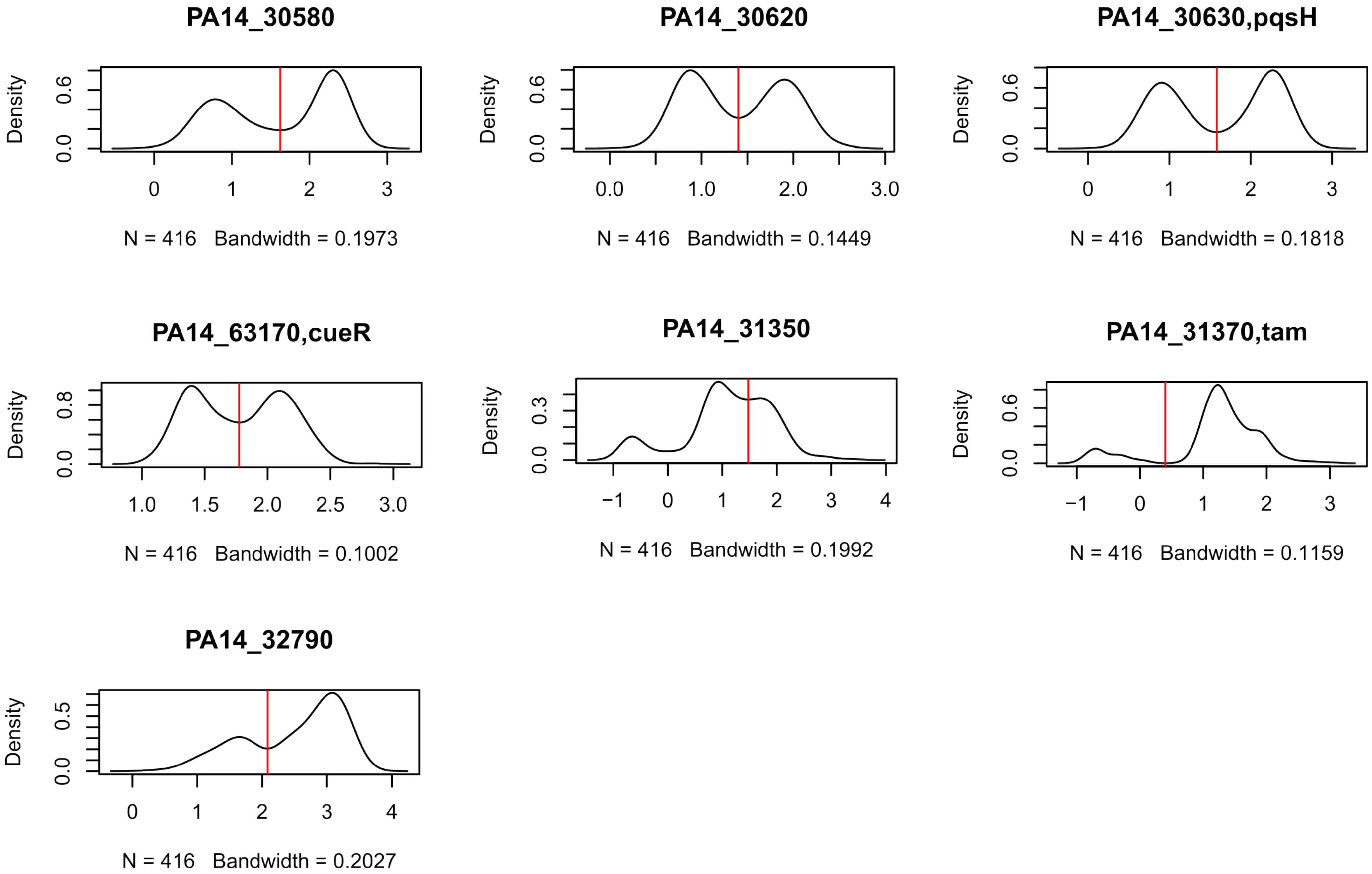
PA14_30580
Density
0.0
0.6
0
1
2
3
N = 416 Bandwidth = 0.1973
PA14_30620
Density
0.0
0.6
0.0
1.0
2.0
3.0
N = 416 Bandwidth = 0.1449
PA14_30630,pqsH
Density
0.0
0.6
0
1
2
3
N = 416 Bandwidth = 0.1818
PA14_63170,cueR
Density
0.0
0.8
1.0
1.5
2.0
2.5
3.0
N = 416 Bandwidth = 0.1002
PA14_31350
Density
0.0
0.3
−1
0
1
2
3
4
N = 416 Bandwidth = 0.1992
PA14_31370,tam
Density
0.0
0.6
−1
0
1
2
3
N = 416 Bandwidth = 0.1159
PA14_32790
Density
0.0
0.5
0
1
2
3
4
N = 416 Bandwidth = 0.2027

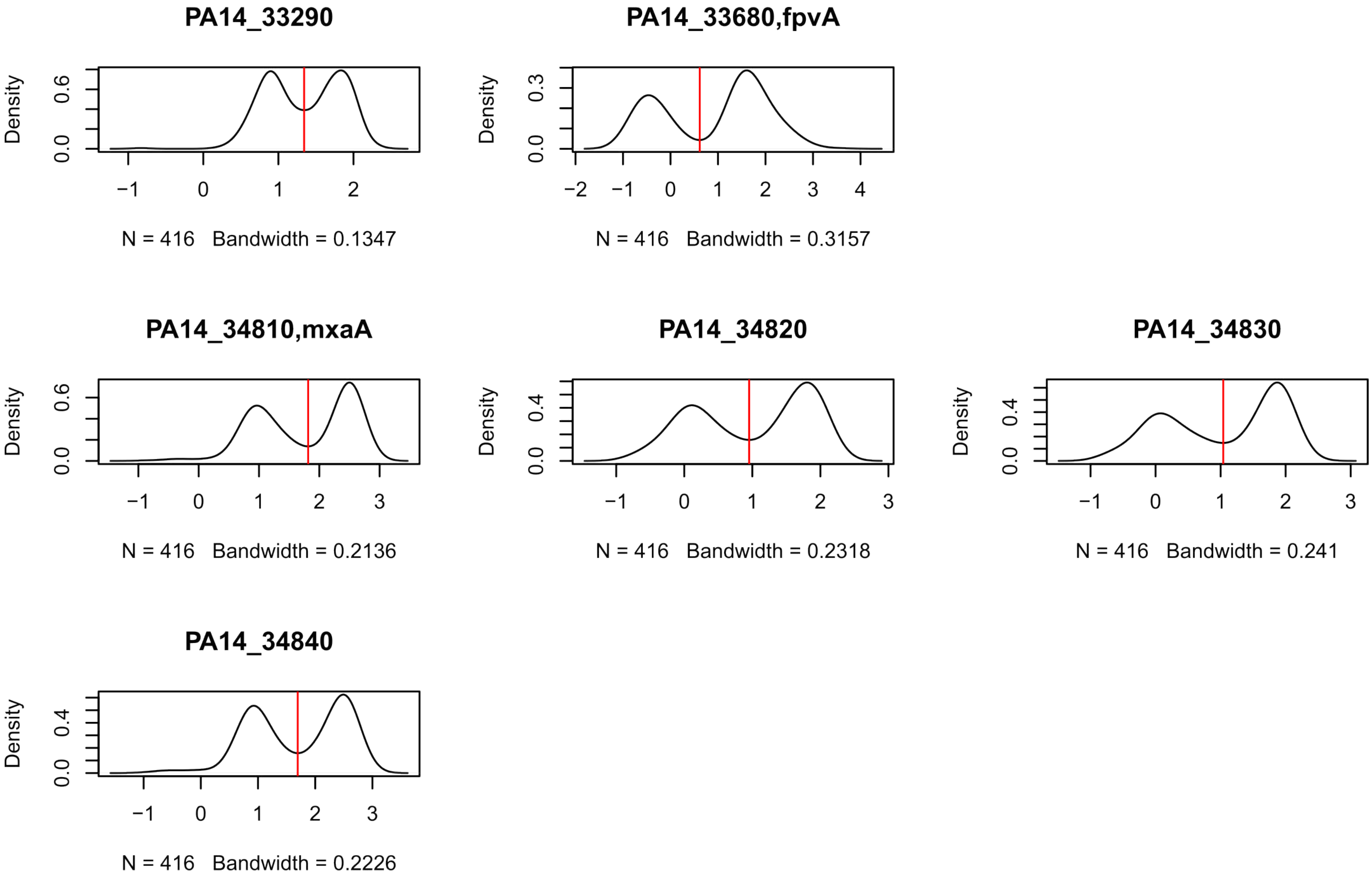
PA14_33290
Density
0.0
0.6
−1
0
1
2
N = 416   Bandwidth = 0.1347
PA14_33680,fpvA
Density
0.0
0.3
−2
−1
0
1
2
3
4
N = 416   Bandwidth = 0.3157
PA14_34810,mxaA
Density
0.0
0.6
−1
0
1
2
3
N = 416   Bandwidth = 0.2136
PA14_34820
Density
0.0
0.4
−1
0
1
2
3
N = 416   Bandwidth = 0.2318
PA14_34830
Density
0.0
0.4
−1
0
1
2
3
N = 416   Bandwidth = 0.241
PA14_34840
Density
0.0
0.4
−1
0
1
2
3
N = 416   Bandwidth = 0.2226

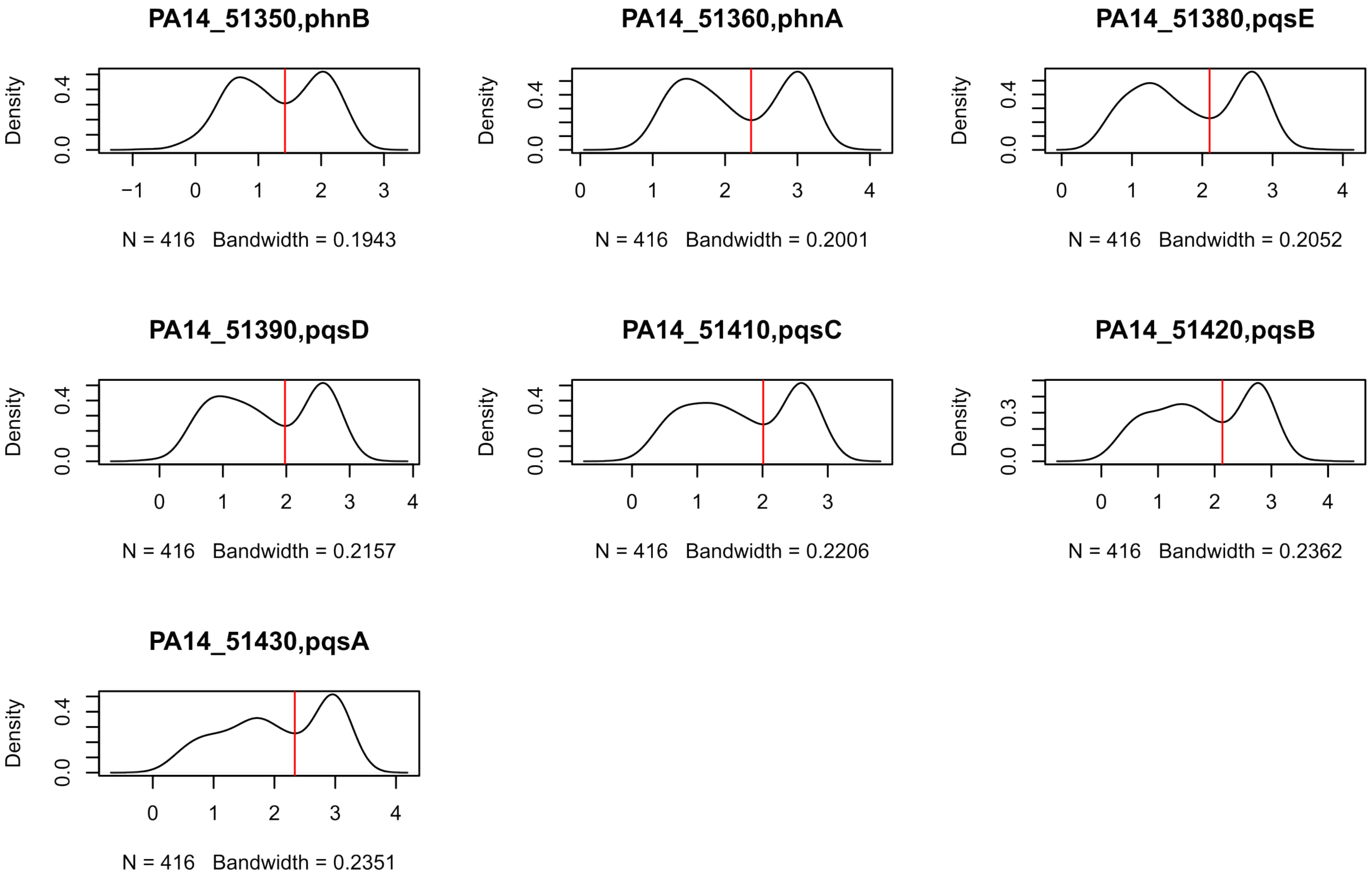
PA14_51350,phnB
Density
0.0
0.4
−1
0
1
2
3
N = 416 Bandwidth = 0.1943
PA14_51360,phnA
Density
0.0
0.4
0
1
2
3
4
N = 416 Bandwidth = 0.2001
PA14_51380,pqsE
Density
0.0
0.4
0
1
2
3
4
N = 416 Bandwidth = 0.2052
PA14_51390,pqsD
Density
0.0
0.4
0
1
2
3
4
N = 416 Bandwidth = 0.2157
PA14_51410,pqsC
Density
0.0
0.4
0
1
2
3
N = 416 Bandwidth = 0.2206
PA14_51420,pqsB
Density
0.0
0.3
0
1
2
3
4
N = 416 Bandwidth = 0.2362
PA14_51430,pqsA
Density
0.0
0.4
0
1
2
3
4
N = 416 Bandwidth = 0.2351

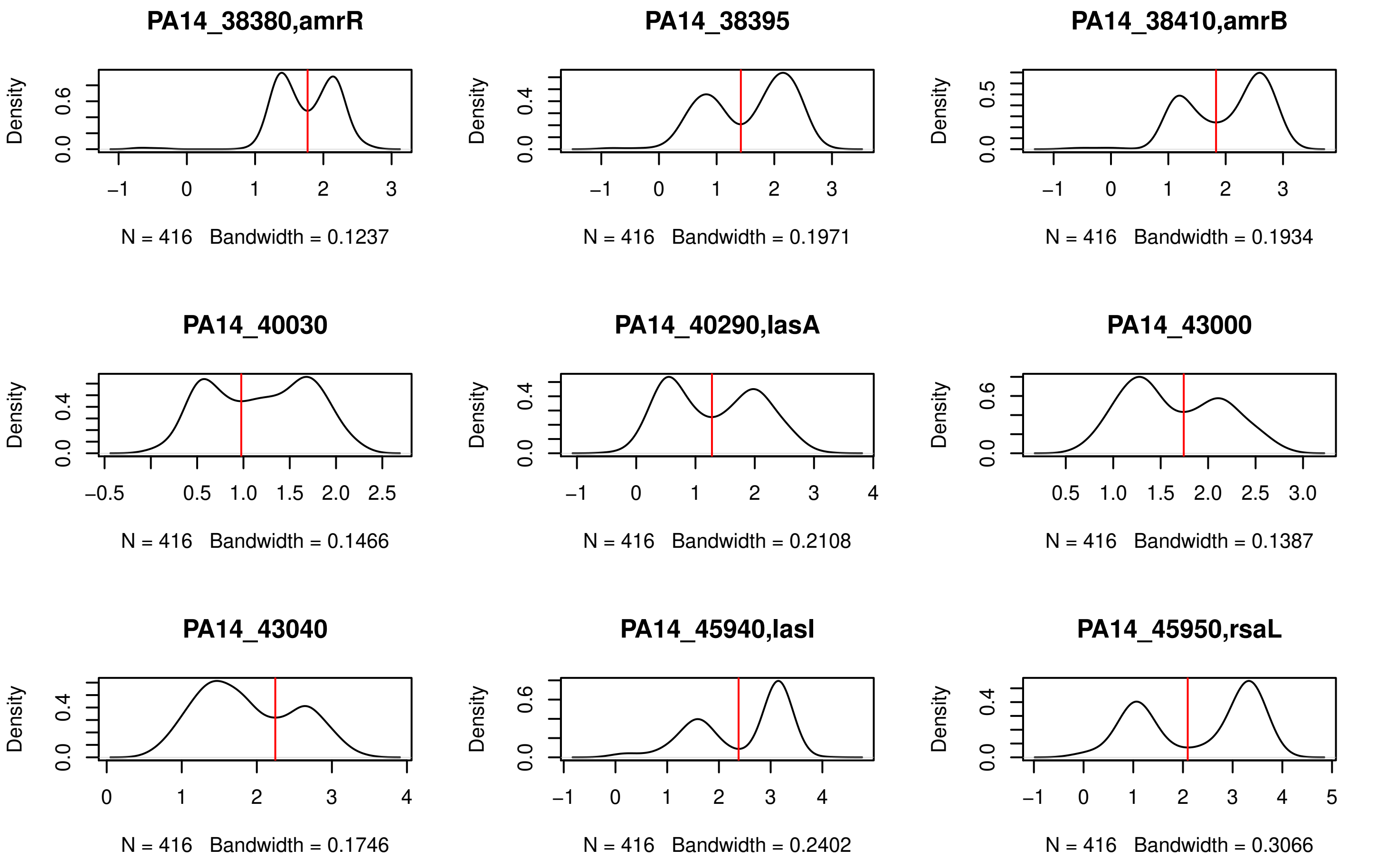
PA14_38380,amrR
Density
N = 416 Bandwidth = 0.1237
PA14_38395
Density
N = 416 Bandwidth = 0.1971
PA14_38410,amrB
Density
N = 416 Bandwidth = 0.1934
PA14_40030
Density
N = 416 Bandwidth = 0.1466
PA14_40290,lasA
Density
N = 416 Bandwidth = 0.2108
PA14_43000
Density
N = 416 Bandwidth = 0.1387
PA14_43040
Density
N = 416 Bandwidth = 0.1746
PA14_45940,lasI
Density
N = 416 Bandwidth = 0.2402
PA14_45950,rsaL
Density
N = 416 Bandwidth = 0.3066

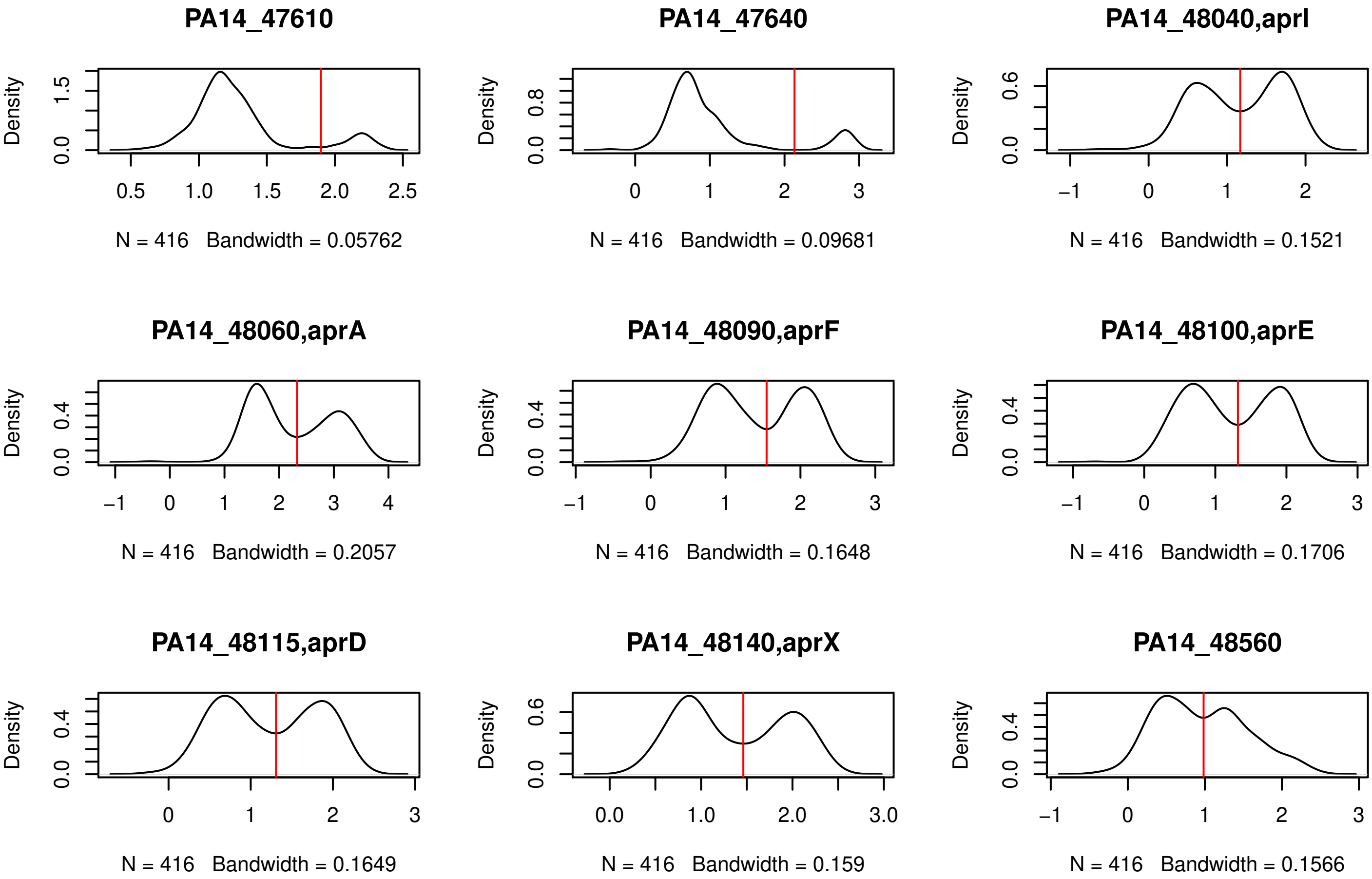
PA14_47610
Density
0.0
1.5
0.5
1.0
1.5
2.0
2.5
N = 416 Bandwidth = 0.05762
PA14_47640
Density
0.0
0.8
0
1
2
3
N = 416 Bandwidth = 0.09681
PA14_48040,aprI
Density
0.0
0.6
−1
0
1
2
N = 416 Bandwidth = 0.1521
PA14_48060,aprA
Density
0.0
0.4
−1
0
1
2
3
4
N = 416 Bandwidth = 0.2057
PA14_48090,aprF
Density
0.0
0.4
−1
0
1
2
3
N = 416 Bandwidth = 0.1648
PA14_48100,aprE
Density
0.0
0.4
−1
0
1
2
3
N = 416 Bandwidth = 0.1706
PA14_48115,aprD
Density
0.0
0.4
0
1
2
3
N = 416 Bandwidth = 0.1649
PA14_48140,aprX
Density
0.0
0.6
0.0
1.0
2.0
3.0
N = 416 Bandwidth = 0.159
PA14_48560
Density
0.0
0.4
−1
0
1
2
3
N = 416 Bandwidth = 0.1566

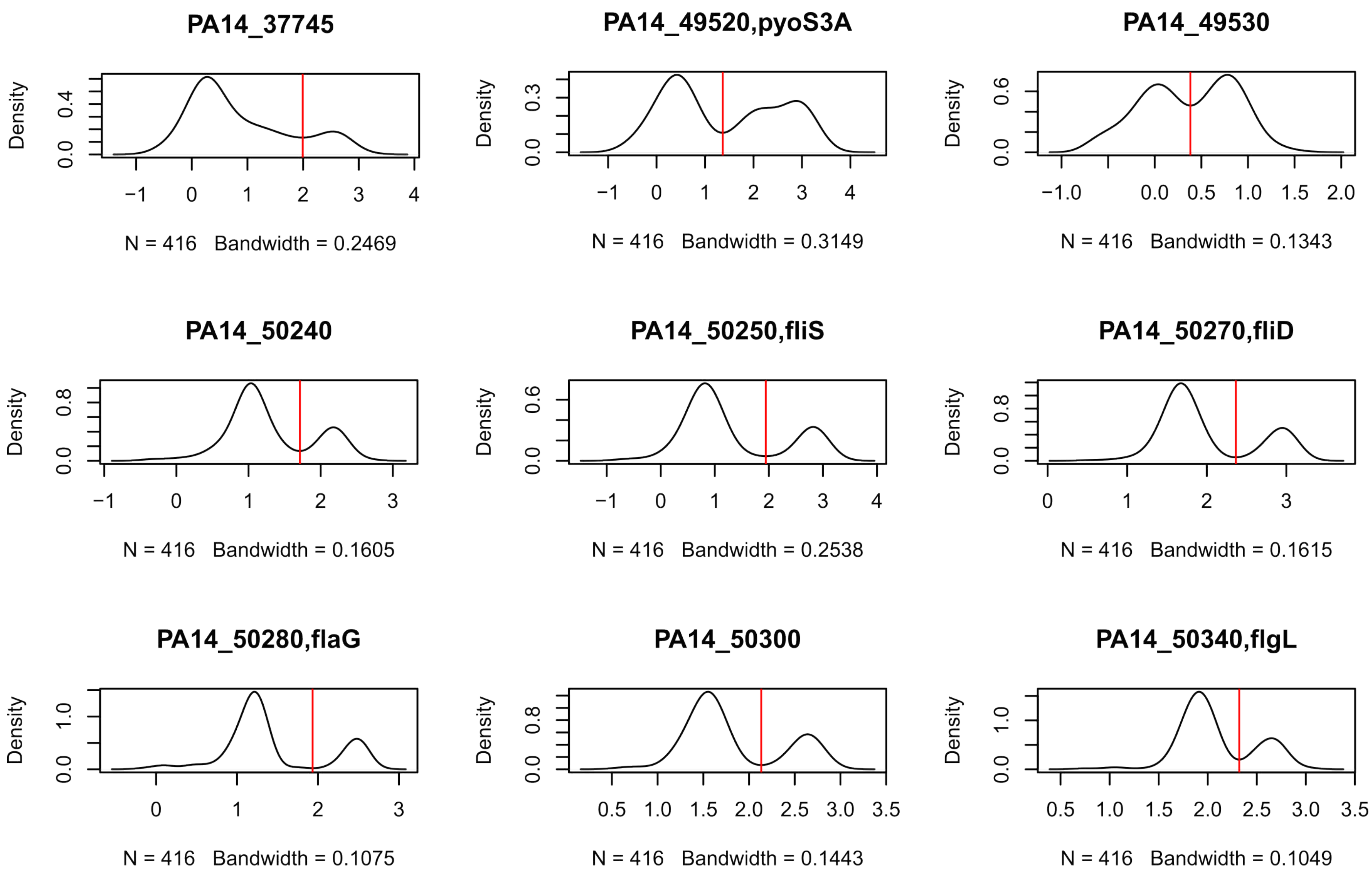
PA14_37745
Density
N = 416 Bandwidth = 0.2469
PA14_49520,pyoS3A
Density
N = 416 Bandwidth = 0.3149
PA14_49530
Density
N = 416 Bandwidth = 0.1343
PA14_50240
Density
N = 416 Bandwidth = 0.1605
PA14_50250,fliS
Density
N = 416 Bandwidth = 0.2538
PA14_50270,fliD
Density
N = 416 Bandwidth = 0.1615
PA14_50280,flaG
Density
N = 416 Bandwidth = 0.1075
PA14_50300
Density
N = 416 Bandwidth = 0.1443
PA14_50340,flgL
Density
N = 416 Bandwidth = 0.1049

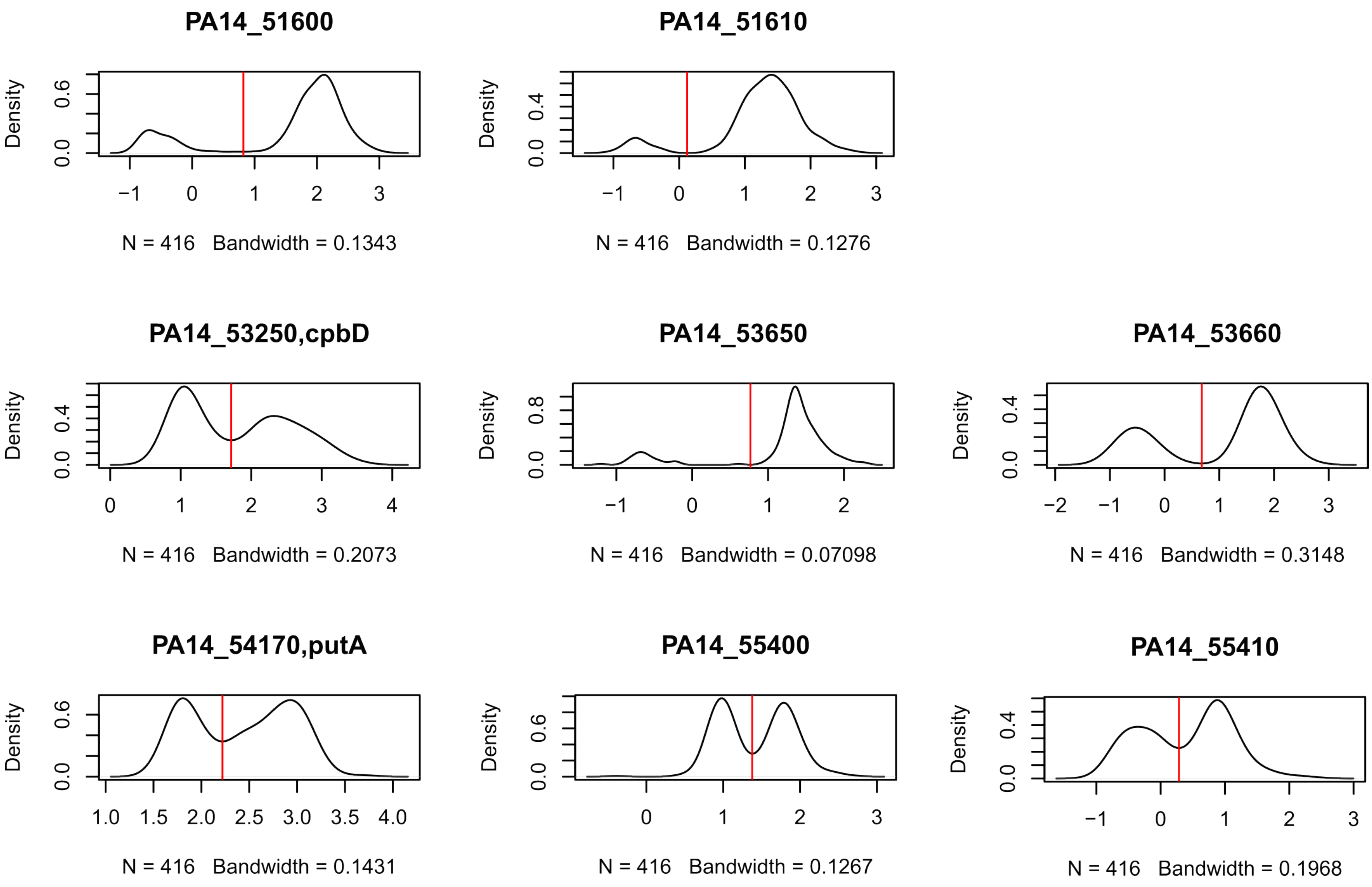

PA14_51600
Density
0.0
0.6
−1
0
1
2
3
N = 416   Bandwidth = 0.1343
PA14_51610
Density
0.0
0.4
−1
0
1
2
3
N = 416   Bandwidth = 0.1276
PA14_53250,cpbD
Density
0.0
0.4
0
1
2
3
4
N = 416   Bandwidth = 0.2073
PA14_53650
Density
0.0
0.8
−1
0
1
2
N = 416   Bandwidth = 0.07098
PA14_53660
Density
0.0
0.4
−2
−1
0
1
2
3
N = 416   Bandwidth = 0.3148
PA14_54170,putA
Density
0.0
0.6
1.0
1.5
2.0
2.5
3.0
3.5
4.0
N = 416   Bandwidth = 0.1431
PA14_55400
Density
0.0
0.6
0
1
2
3
N = 416   Bandwidth = 0.1267
PA14_55410
Density
0.0
0.4
−1
0
1
2
3
N = 416   Bandwidth = 0.1968

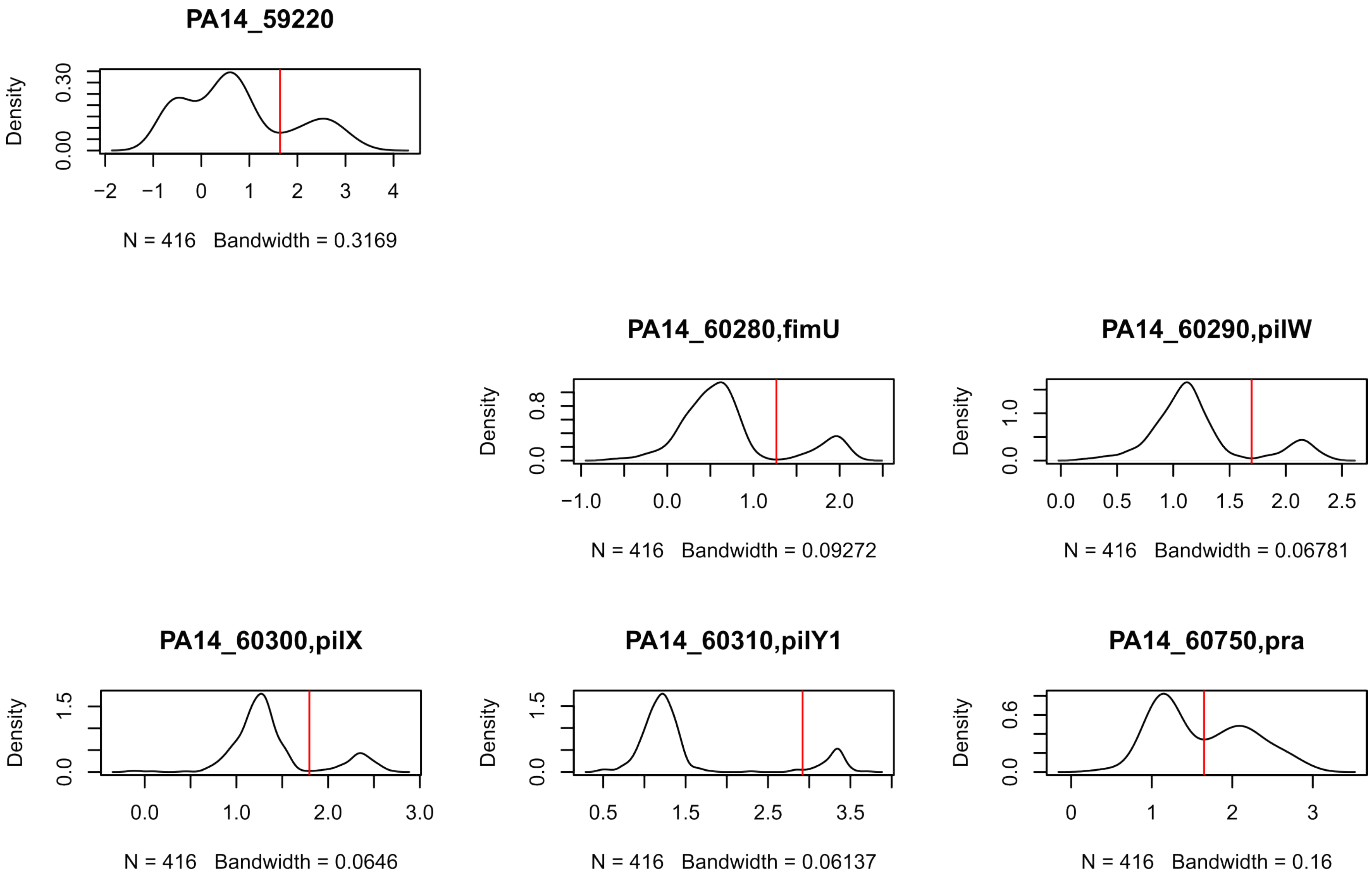
PA14_59220
Density
0.00
0.30
−2
−1
0
1
2
3
4
N = 416 Bandwidth = 0.3169
PA14_60280,fimU
Density
0.0
0.8
−1.0
0.0
1.0
2.0
N = 416 Bandwidth = 0.09272
PA14_60290,pilW
Density
0.0
1.0
0.0
0.5
1.0
1.5
2.0
2.5
N = 416 Bandwidth = 0.06781
PA14_60300,pilX
Density
0.0
1.5
0.0
1.0
2.0
3.0
N = 416 Bandwidth = 0.0646
PA14_60310,pilY1
Density
0.0
1.5
0.5
1.5
2.5
3.5
N = 416 Bandwidth = 0.06137
PA14_60750,pra
Density
0.0
0.6
0
1
2
3
N = 416 Bandwidth = 0.16

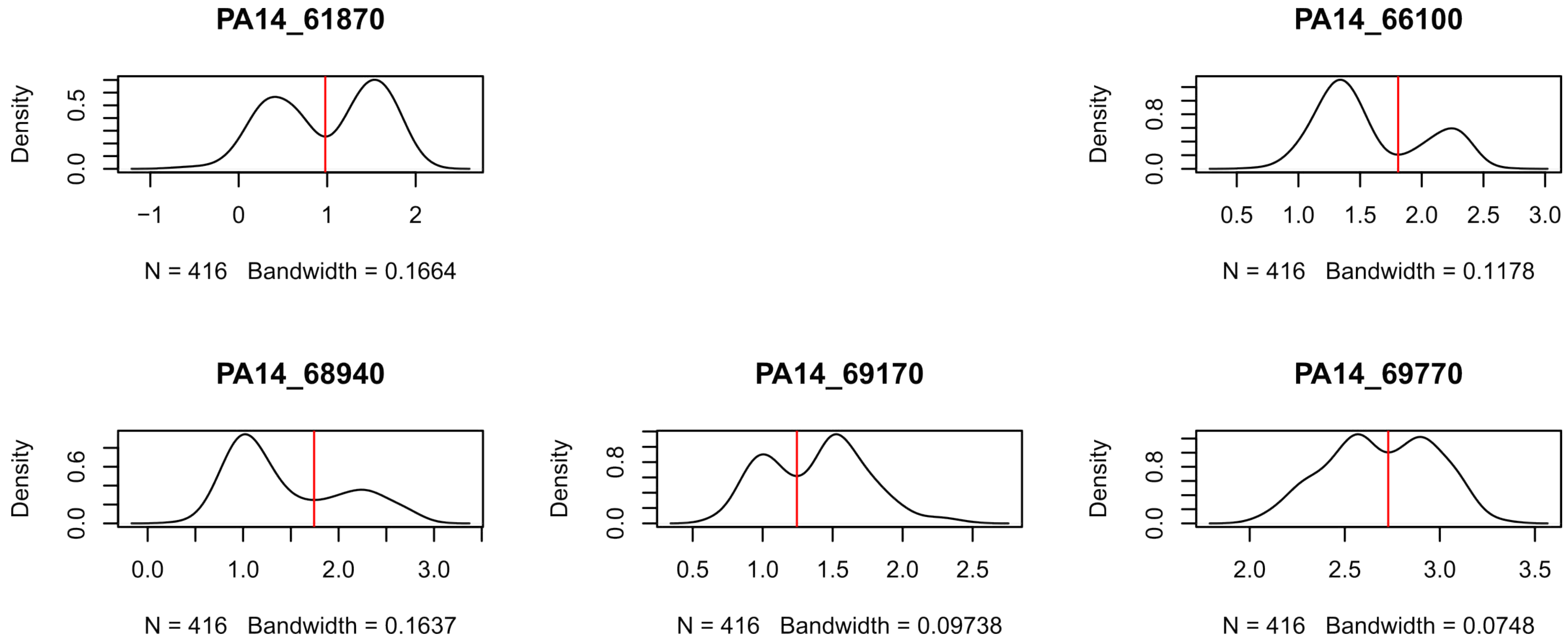
PA14_61870
Density
0.0
0.5
−1
0
1
2
N = 416 Bandwidth = 0.1664
PA14_66100
Density
0.0
0.8
0.5
1.0
1.5
2.0
2.5
3.0
N = 416 Bandwidth = 0.1178
PA14_68940
Density
0.0
0.6
0.0
1.0
2.0
3.0
N = 416 Bandwidth = 0.1637
PA14_69170
Density
0.0
0.8
0.5
1.0
1.5
2.0
2.5
N = 416 Bandwidth = 0.09738
PA14_69770
Density
0.0
0.8
2.0
2.5
3.0
3.5
N = 416 Bandwidth = 0.0748

Supplementary Table

Supplementary Table 1: Oligonucleotides used in this study

Name	Sequence (5' - 3')	Goal of the primer	Other characteristics
rhlR_PA14_spc2_F	**gcgcg**GGCTTCGATTACTACGCCTA	Oligonucleotide in the sense orientation for the construction of the *rhlR*-targeting sgRNA	The nt depicted as bold characters were added to reconstruct the Eco31I protruding ends after plasmid hibridization for cloning in pS448·CsR
rhlR_PA14_spc2_R	**aaac**TAGGCGTAGTAATCGAAGCC**c**	Oligonucleotide in the antisense orientation for the construction of the *rhlR*-targeting sgRNA	The nt depicted as bold characters were added to reconstruct the Eco31I protruding ends after plasmid hibridization for cloning in pS448·CsR
phoB_PA14_spc2_F	**gcgcg**ACGGCGATCATCTCGCGAAT	Oligonucleotide in the sense orientation for the construction of the *phoB*-targeting sgRNA	The nt depicted as bold characters were added to reconstruct the Eco31I protruding ends after plasmid hibridization for cloning in pS448·CsR
phoB_PA14_spc2_R	**aaac**ATTCGCGAGATGATCGCCGT**c**	Oligonucleotide in the antisense orientation for the construction of the *phoB*-targeting sgRNA	The nt depicted as bold characters were added to reconstruct the Eco31I protruding ends after plasmid hibridization for cloning in pS448·CsR
rhlR_PA14(+)-Rec	cgggccaattctgctgtgatgcattttatcgatcagg gcttactgca**ATGTGA**agcgtagggcgcgccg gccggcgcgccctaccagatctggcaggttg	Recombineering oligonucleotide used for the deletion of *rhlR* in PA14	The start (ATG) and stop codons of *rhlR* are depicted as bold characters
phoB_PA14(+)-Rec	tttcgttatctaatgcggcgcaagacagatcgaccc gaggcaagacc**ATGTGA**ccccgcgcccggc cgcccggcggccggagtcgtttcccctggacggt	Recombineering oligonucleotide used for the deletion of *phoB* in PA14	The start (ATG) and stop codons of *phoB* are depicted as bold characters
rhlR_PA14-Seq-F	CTGTCGGCGTTTCATGGAATTG	Forward primer used for PCR and sequencing of the *rhlR* deletion	Tm = 58ºC
rhlR_PA14-Seq-R	AAGACTTGATGCCGGTAGCGTC	Reverse primer used for PCR and sequencing of the *rhlR* deletion	Tm = 60ºC
phoB_PA14-Seq-F	CGCCAGGTGGATTCTGTTCATCG	Forward primer used for PCR and sequencing of the *phoB* deletion	Tm = 61ºC
phoB_PA14-Seq-R	GCGTTGCAGGTGGTAGATATTG	Reverse primer used for PCR and sequencing of the *phoB* deletion	Tm = 57ºC

aaSSR-1F	TGAGCCAAGTAGCCAGGGTCG	Forward primer used to check the prescence of *ssr* gene by PCR	Tm = 60ºC
aaSSR-1R	GATTTGTAGACGTTGGCAGCGCAG	Reverse primer used to check the prescence of *ssr* gene by PCR	Tm = 60ºC
aaCas9-1F	AAGCACAAGTGTCTGGACAAGG	Forward primer used to check the prescence of *S. pyogenes cas9* gene by PCR	Tm = 58ºC
aaCas9-1R	TCACTCAAACCTCCACGTTCAGC	Reverse primer used to check the prescence of *S. pyogenes cas9* gene by PCR	Tm = 58ºC

Supplementary Data

Supplementary Data 1: Bimodally expressed genes and their affiliation to clusters containing genes with similar expression patterns across clinical isolates. KEGG pathways were assigned using the R package KEGGREST (version 1.26.1).

Supplementary Data 2: Genes that are significantly (FDR ≤ 0.05) and relevantly ($log_2FC \geq 1$; $log_2FC \leq -1$) differentially expressed in clinical *lasR** compared to *lasR*^{WT} isolates under planktonic growth conditions ($OD_{600} = 2$).

Supplementary Data 3: Genes that are significantly (FDR ≤ 0.05) and relevantly ($log_2FC \geq 1$; $log_2FC \leq -1$) differentially regulated between PA14Δ*lasR* compared to PA14 WT under planktonic growth conditions ($OD_{600} = 2$).

Supplementary Data 4: Genes that are significantly (FDR ≤ 0.05) and relevantly ($log_2FC \geq 1$; $log_2FC \leq -1$) differentially regulated between clinical *lasR** compared to *lasR*^{WT} isolates under biofilm growth conditions (48h growth in LB at 37°C).

Supplementary Data 5: Genes that are significantly (FDR ≤ 0.05) and relevantly ($log_2FC \geq 1$; $log_2FC \leq -1$) differentially regulated between PA14Δ*lasR* compared to PA14 WT under biofilm growth conditions (48h growth in LB at 37°C).

Supplementary Data 6: **Strain-specific as well as common transcriptional signatures associated with *lasR* deficiency.** Genes (PA14 Locus Tags) that are expressed below $log_2FC = -1$ in the respective number of *lasR** isolates compared to all *lasR*^{WT} isolates (sheet "lasRmut vs all lasRWT down"). Genes (PA14 Locus Tags) that are expressed above $log_2FC = 1$ in the respective number of *lasR** isolates compared to all *lasR*^{WT} isolates (sheet "lasRmut vs all lasRWT up").

Supplementary Data 7: Differential gene expression between biofilm and planktonic growth in clinical isolates harboring a *lasR* wildtype allele or a lasR deficiency. Genes that are significantly (FDR ≤ 0.05) and relevantly ($log_2FC \geq 1$; $log_2FC \leq -1$) differentially expressed in clinical *lasR*^{WT} under planktonic growth conditions ($OD_{600} = 2$) compared to *lasR*^{WT} isolates under biofilm growth conditions (48h growth in LB at 37°C) (sheet "lasR WT PL vs BF"). Genes that are significantly (FDR ≤ 0.05) and relevantly ($log_2FC \geq 1$; $log_2FC \leq -1$) differentially expressed in clinical *lasR** isolates under planktonic growth conditions ($OD_{600} = 2$) compared to *lasR** isolates under biofilm growth conditions (48h growth in LB at 37°C) (sheet "lasR Mutants PL vs BF").
